# Distinct motor preparatory signals correlate with coarse *vs* fine control of self-paced action timing

**DOI:** 10.1162/IMAG.a.1434

**Published:** 2026-11-04

**Authors:** Silvia Seghezzi, Elisabeth Parés-Pujolràs, Patrick Haggard

**Affiliations:** Institute of Cognitive Neuroscience, University College London, London, United Kingdom; Birkbeck, University of London, London, United Kingdom; University College Dublin, Belfield, Dublin, Ireland

**Keywords:** motor timing, learning, action planning, readiness potential, beta-band EEG power

## Abstract

Making each action at the right time is crucial for motor control. In this study, we investigated how two prominent motor preparatory processes identified in human EEG—beta-band desynchronisation and the readiness potential (RP)—might each differently relate to the time of a simple voluntary action. In our paradigm, participants learned when to act in each block of trials, based on probabilistic trial-by-trial feedback. A reward schedule reinforced a specific optimal time to act in some blocks, but allowed greater variability around the same mean action time in other blocks. We found that contralateral motor beta-band power at the beginning of each trial was higher in trials with slow actions than in those with fast actions. In contrast, contralateral beta-band power reached stereotyped low values at the time of action, regardless of waiting times. Put together, this pattern is consistent with a neural implementation of distance-to-bound for a noisy accumulation process, in which baseline motor activity starts off closer to an action-triggering bound when fast actions are required. In turn, the RP was independent of the mean waiting time before action, but its slope increased when participants developed a precise motor plan. We suggest that beta desynchronisation and the RP track distinct, complementary processes involved in the temporal control of action. While beta-power activity at trial onset is consistent with a strategic adjustment of motor preparation to match the required pace of action, the modulation of the RP by motor plan precision is consistent with a temporally structured input to a ramping preparatory process. Simulations suggest that acting at the right time may involve increasing the strength of the input to this accumulator, which can drive the decision variable to drift towards the decision bound.

## 1 Introduction

The ability to precisely time our actions is essential for successful voluntary motor control. Humans are able to set extremely precise internal timers, synchronising their actions to events in the external world with up to millisecond accuracy (Merchant et al., 2008). Expert musicians and dancers routinely perform a movement at exactly the time required, or at exactly the time of their choosing, even in the absence of external cues. This implies the presence of an internal go signal—a neural process that enables self-paced action based on internally generated information, rather than external triggers. The neural events preceding simple self-generated actions have been studied by a wide body of research in humans and other species. These studies have identified circuits that specify both whether and also when to act in a given context (de Lafuente et al., 2024; Hoffstaedter et al., 2013; Khalighinejad, Bongioanni, et al., 2020; Khalighinejad, Priestley, et al., 2020; Krieghoff et al., 2009; Zapparoli et al., 2017, 2018). However, the neural mechanisms that contribute to decisions about action timing are not fully understood. For example, actions that are strategically delayed, as in prospective memory or postponed by self-restraint (Mischel et al., 1972), might depend on some as-yet unknown internal go signal.

Several kinds of computational models have been put forward to describe how temporal decisions are made (Paton & Buonomano, 2018). Amongst these, ramping models have been particularly successful at explaining both behavioural and neural patterns of activity in self-paced actions. Ramping models propose that time is encoded through gradual, monotonic changes in neural activity resulting from the accumulation of tonic neural input. The accumulating signal triggers a decision when the corresponding neural code reaches a threshold or bound (Durstewitz, 2003; Paton & Buonomano, 2018; Simen et al., 2011). These models are thus similar to accumulation-to-bound models of perceptual decision making (O’Connell et al., 2012; Ratcliff & McKoon, 1988; Ratcliff et al., 2016), where the accumulation of external sensory input up to a bound can account for the observed behavioural and neural patterns across species (Balcı & Simen, 2016; Merchant & Averbeck, 2017; O’Connell et al., 2012; Shadlen & Kiani, 2013; Simen et al., 2016). This framework distinguishes between the accumulation process, which governs the shape and timing of the ramp-like activity observed across several brain areas, and the decision threshold, which determines when the process terminates in action.

In studies of perceptual decision making in humans, these two aspects of the decision process have been linked to distinct neural mechanisms. First, a progressive increase in mean EEG amplitude over centroparietal areas tracks abstract (i.e., effector-independent) accumulation of sensory evidence (O’Connell et al., 2012). Second, contralateral motor beta-band power decreases progressively during motor preparation, reaching a stereotyped low minimum at the time of action. This minimum is invariant across action latencies and task conditions, and has, therefore, been interpreted as a fixed action-triggering threshold (Kelly et al., 2021; O’Connell & Kelly, 2021). In the case of purely self-paced actions, there may be no sensory evidence to accumulate. Instead, the accumulating signals are assumed to be internally generated. However, the distinction between accumulation and strategic bounds remains important also for voluntary, self-paced action. In this study, we investigate two neural precursors of movement that may be related to these two aspects of decision: the readiness potential (RP) and motor beta desynchronisation (beta-ERD).

The RP is a slow-building negative deflection observed before voluntary movements (Deecke et al., 1969, 1987; Kornhuber & Deecke, 1965) that shifts towards the hemisphere contralateral to the limb that actually moves just prior to movement onset (Haggard & Eimer, 1999; O’Connell et al., 2012). The RP is believed to originate in the pre-supplementary motor areas (Cui & Deecke, 1999; Erdler et al., 2000; Fried et al., 2011; Gavenas et al., 2024; Praamstra et al., 1996; Seghezzi et al., 2019; Toma et al., 2002)—areas which also play a role in performance monitoring and action control (Ullsperger et al., 2014). The average RP shape is similar to the ramping increase of single-cell firing rates in motor areas of non-human primates (Romo & Schultz, 1987) and rodents (Murakami et al., 2014, 2017) prior to movement. Further, a specific implementation of a ramping model, namely the stochastic accumulator model (Schurger et al., 2012), suggests that the characteristic shape of the average RP is simply a by-product of averaging multiple instances of noisy accumulation processes with a fixed, temporally invariant input that is assumed to capture a general level of urgency. While the shape and amplitude of the RP do not seem to be under direct voluntary control (Schultze-Kraft et al., 2021), one recent study showed that the RP shape was sensitive to the precision with which participants learned from feedback when to make an action (Travers et al., 2021). In particular, a stronger RP was associated with a more precise plan of when to act. This suggests that, even if the RP captures a noisy accumulation process, the input to such accumulation process may depend on processes related to temporal cognition.

In turn, beta-ERD refers to a gradual reduction in EEG power in the beta (13–30 Hz) frequency range, which has been routinely observed during motor preparation during perceptual decision making (e.g., Kelly et al., 2021) and self-paced spontaneous actions (Pfurtscheller & Lopes da Silva, 1999; Stancák & Pfurtscheller, 1996). Beta power reaches a minimum at the time of action over the motor cortex contralateral to movement (McCone, 2024; McCone et al., 2024; Murphy et al., 2016; O’Connell et al., 2012; Steinemann et al., 2018), even if the action latency from start of trial varies considerably (Kelly et al., 2021). In turn, changes in motor beta power at the beginning of evidence accumulation in perceptual decision-making tasks are associated with speed–accuracy trade-offs (Kelly et al., 2021; Kirschner et al., 2024; Steinemann et al., 2018). Trial-by-trial adjustments to baseline levels of motor beta power have also been observed in time interval reproduction tasks (McCone et al., 2024). Further, neurofeedback experiments have shown that agents are able to voluntarily control motor beta-ERD (He et al., 2020). Computationally, this evidence suggests that motor activity at the start of decision-making processes can be strategically lowered or increased, adjusting the distance that a motor preparation process needs to cover to hit an action-triggering bound, thus regulating average action times (Kelly et al., 2021).

In summary, while action preparation is typically accompanied by both an RP and by beta-ERD, these may reflect distinct aspects of the motor planning process (Parés-Pujolràs et al., 2023). In particular, the RP may represent an accumulating decision variable for self-paced action, while contralateral motor beta-band power may index the distance of the motor preparation process from a fixed action-triggering bound—a distance that can be strategically adjusted at trial onset to produce faster or slower actions. However, an integrated account of how the two signals may jointly determine exactly when a self-paced action occurs remains missing.

Here, we designed a reinforcement learning paradigm to allow participants to learn the time of a self-paced action. The behavioural paradigm was designed to allow the respective roles of RP and motor beta power signals to be identified. The reinforcement schedule and the optimal time to act were manipulated between blocks. One schedule required participants to develop a very precise motor timing plan, while another schedule yielded high reward rates even if action timing varied substantially. Crucially, we orthogonally manipulated the mean target waiting time, so that participants had to act after a short delay in some blocks, but after a much longer delay in other blocks. This experimental design thus independently varies the “urgency” to act—that is, how fast or slow actions need to be—and the precision with which target times must be reached in order to obtain a reward. By orthogonally manipulating these two factors, our design potentially allows for a functional dissociation between neural markers such as RP and beta-band power. Based on previous findings (Travers et al., 2021), we hypothesised that the shape of the RP would reflect motor plan precision, with RP slope increasing once participants learned the correct time to act in each block. Conversely, we hypothesised that motor beta power around trial start would reflect urgency-related modulations of baseline motor preparation, and would, therefore, depend on participants’ estimates of the mean target time in each block.

## 2 Materials and Methods

### 2.1 Participants

Participants were recruited from the UCL psychology subject pool. All participants were healthy, aged between 18 and 35 years, right handed, fluent in English, with normal or corrected-to-normal vision, no known disabilities, and no history of neurological or psychological disorder.

In total, 25 participants (mean ± SD: 24.0 ± 3.8 years) took part in the experiment. Our sample size was guided by the only closely related study that has examined the relationship between trial-by-trial changes in waiting time and readiness potential (RP) amplitude (Travers et al., 2021). That study reported a significant linear mixed-model effect of |δWT| (in seconds) on RP amplitude (in μV), b = −0.29, CI [0.02, 0.56], p = 0.038, SD across subjects = 0.16, and detected this effect with 19 participants. Because we could not reproduce an exact mixed-model power analysis, we used G*Power to obtain an approximate benchmark, modelling our design as a multiple linear regression with two predictors (test family: F tests; statistical test: linear multiple regression, fixed model, R² deviation from zero; α = 0.05, two-tailed). For a fixed sample size of N = 25, this analysis indicates that the study would achieve around 80% power for large combined effects of the predictors (approximately Cohen’s f² ≈ 0.45, corresponding to R ≈ 0.56).

### 2.2 Procedure

The experiment was approved by the UCL ICN ethics committee (ICN-PH-PWB-20-02-2018A).

All participants were invited to a single EEG session.

Written informed consent was obtained from all participants before beginning the experiment.

Participants sat in a quiet room and viewed the stimuli on a computer monitor at 50 cm distance. Instructions were displayed on the computer screen. Before starting the experiment, instructions were verbally repeated by the experimenter.

Participants received a £7.50 per hour reimbursement upon completing the experiment, plus a bonus depending on their performance.

### 2.3 Task

The experiment was programmed in Matlab R2014b using the Psychophysics Toolbox-3 extensions (Brainard, 1997; Kleiner et al., 2007; Pelli, 1997).

The task consisted of a modified version of the temporal reinforcement learning paradigm developed by Travers et al. (2021), adapted into a “farmer” cover story previously used by Seghezzi et al. (2026), where participants were asked to learn the best time to make a voluntary keypress action using the information provided by a supervisory feedback signal. Each block was associated with one of five optimal waiting times (2.25/3.5/4.75/6.0/7.25), and feedback was probabilistically determined by the same reward window (narrow/wide) throughout each block (see below). These two experimental factors (target time and reward window) allowed us to dissociate the general urgency to act (target time) from the precision of the motor plans participants developed in each block (reward window).

Participants acted as “farmers”, waiting to harvest a seed that was “planted” at the beginning of each trial. Their task was to wait until they believed that the plant was ready for harvesting. Each trial started with the seed being planted in the ground (“Planting…”). After 1 s, a fixation cross appeared in the centre of the screen. At any self-chosen time thereafter, participants could press the spacebar with their right hand to harvest the plant from the ground; the maximum waiting time allowed per trial was 15 s. If participants harvested the plant at the right time, they were likely to gain a small extra cash bonus (£0.01). If they harvested the plant before it was ready or too late, they were likely to lose a small cash amount (£-0.01). Following a 1 s delay after the keypress, feedback was provided for 1 s as a visual display containing a “Too early!”/ “Perfect!”/ “Too late!” message, the harvested plant, and a notice with the monetary loss/win (£±0.01) (see Fig. 1A).

**Fig. 1.**
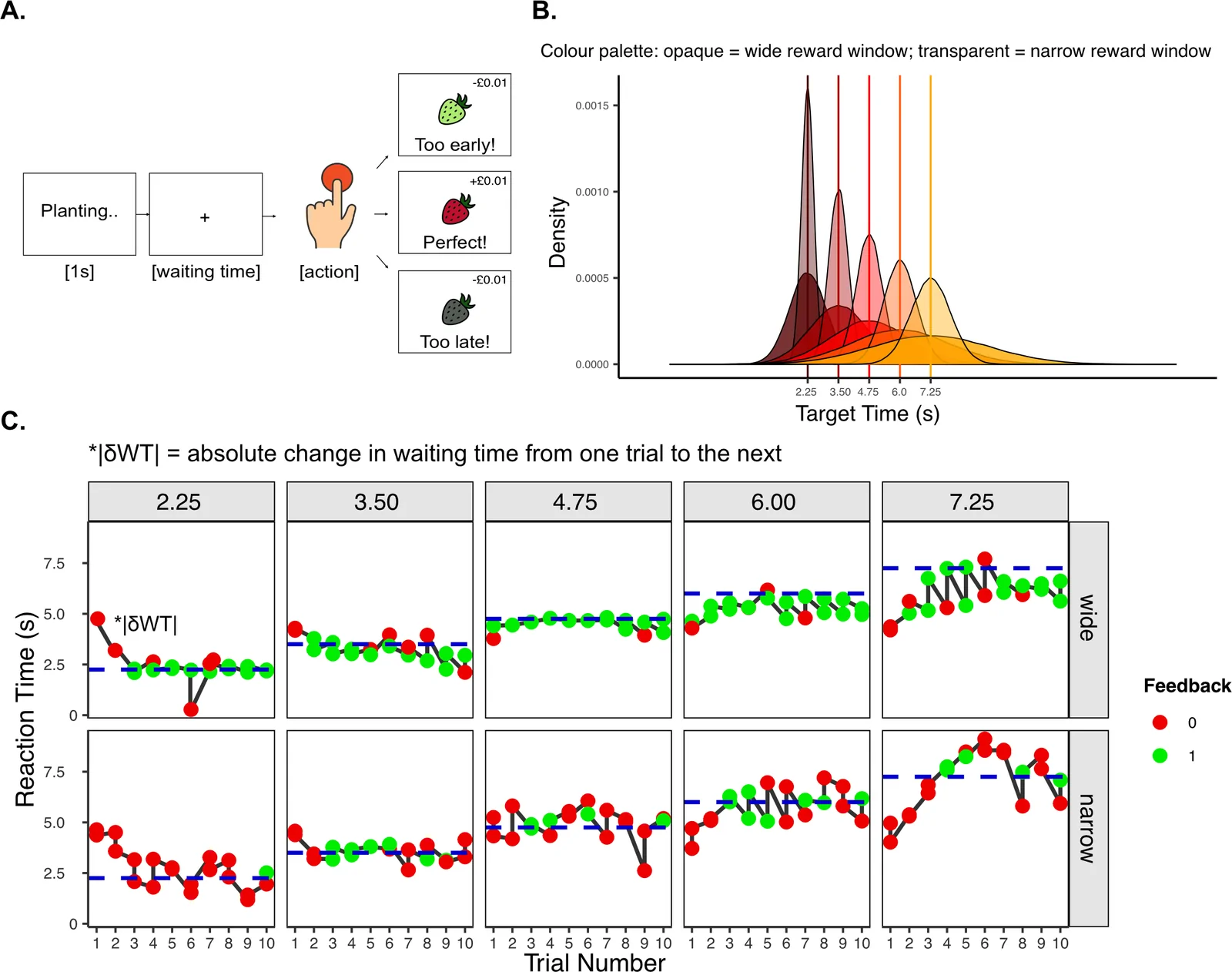
Experimental Task Overview, Reward Windows around Target Times, and Single‐Subject Block Data. (A) Experimental task. Participants (N = 25) learnt the correct waiting time to “harvest” a plant or vegetable from the ground by receiving performance feedback. (B) Density plots of reward windows around target times (2.25, 3.5, 4.75, 6, and 7.25 s) in each block, with opaque curves representing the wide reward window condition and more transparent curves indicating the narrow condition. Vertical lines indicate target times. (C) Data for a single participant in one block, where waiting times are plotted across trials; a continuous black line traces the waiting time trend, while individual trial points are coloured red (feedback 0, negative (too early or too late)) or green (feedback 1, positive).

Trial-by-trial feedback was probabilistically drawn from a Gaussian distribution that defined the reward window around each block’s target time. This distribution could be either narrow (i.e., low standard deviation) or wide (i.e., high standard deviation). Given that temporal estimation becomes less precise as duration increases, following Weber’s law (Gibbon, 1977; Gibbon et al., 1997; Haigh et al., 2021; Merchant et al., 2008), the reward windows were computed to match the expected precision for each target time. Specifically, the standard deviations of narrow and wide reward windows for each target time were set to either 11% or 33% of the target time, respectively:

- Target 2.25 s: 250 ms/750 ms
- Target 3.5 s: 389 ms/1167 ms
- Target 4.75 s: 528 ms/1583 ms
- Target 6.0 s: 667 ms/2000 ms
- Target 7.25 s: 806 ms/2417 ms

Thus, actions happening at latencies within ±11%/33% of the target time had a higher probability of receiving positive feedback. Actions with larger temporal errors had progressively lower probabilities of positive feedback (see Fig. 1B).

For each actual response by the participant, a standardised (Z) score was computed as (waiting time – target time) divided by the standard deviation of the reward window in that particular block. The Z score served then as an input to compute the associated probability of having “correct” feedback according to a two-tailed Gaussian tail model, using the formula P(correct) = 2 × (1 − Φ(|Z|)), where Φ is the standard normal cumulative distribution function (so that identical positive and negative deviations from the target yield the same probability). For example, in a narrow-reward block with SD = 250 ms and a target of 2.25 s, a response 300 ms too late (i.e., at 2.55 s after “planting”) would produce Z = 0.30/0.25 = 1.2. The two-tailed tail probability beyond ±1.2 SD of the target is 0.23; therefore, the model assigns a 23% chance of “correct” feedback (in this example, the error message would read “Too late!”). In contrast, in a wide-reward block with SD = 750 ms, exactly the same time of responding produces Z = 0.3/0.75 = 0.4, yielding a much higher probability (≈69%) of “correct” feedback. This example illustrates how the same error in deciding when to act can lead to different feedback depending on the reward window. A wider reward window implied greater tolerance towards temporally imprecise button presses: for any given actual response time, participants would be more likely to receive positive feedback in the wide reward window condition. However, this feedback would be less informative for adapting future behaviour than in the narrow reward window condition, as it made it more difficult for participants to build a precise representation of the optimal action time.

Before starting the experiment, participants completed one block to get familiar with the task and check their understanding. In these training blocks, the optimal waiting time to be learned was 3 s, and the reward window of the feedback probability distribution was 500 ms. During the experiment, each block started with the presentation of the plant to be harvested, and pictures of the unripe, ripe, and over-ripe plant were shown. The unripe plant was coloured green; the ripe plant was coloured according to its natural colour; and the over-ripe plant was coloured grey. Participants completed 20 blocks in total, provided by the factorial combination of mean target time (2.25/3.5/4.75/6.0/7.25 s) and Feedback Reward window (narrow/wide), repeated twice. The blocks were presented in a randomised order. Each block consisted of 10 trials, giving a total of 200 trials for each participant. In sum, our 2 x 5 design factorially manipulated motor precision and urgency, and comprised a total of 100 trials in each reward window condition (narrow/wide), and 40 repetitions in each of the 5 target times.

### 2.4 EEG recording

EEG was recorded from 26 scalp sites (FZ, FCZ, CZ, CPZ, PZ, POZ, FC1, FC2, C1, C2, CP1, CP2, F3, F4, F7, F8, C3, C4, CP5, CP6, FC5, FC6, P3, P4, O1, O2) using active electrodes (g.LADYbird) fixed to an EEG cap (g.GAMMAcap) according to the extended international 10/20 system. EEG data were acquired using a g.GAMMAbox and g.USBamp with a sampling frequency of 256 and 0.01 Hz high-pass and 100 Hz low-pass online filters. Signal was recorded using g.Recorder (G.tec, medical engineering GmbH, Austria). All electrodes were online referenced to the right ear lobe. Vertical and horizontal electrocular activity was recorded from electrodes above and below the right eye and on the outer canthi of both eyes.

### 2.5 Data analysis

#### 2.5.1 Behavioural data analysis

***Variables of interest:*** Following Travers et al. (2021), we quantified learning on a trial-by-trial basis using |δWT|, the absolute change in waiting time from one trial to the next. This metric is particularly useful for capturing each participant’s transition from an exploratory phase (characterised by big |δWT|) to an exploitative phase (with small |δWT|). It was proposed as a more sensitive measure of updating than trial number alone or timing error. In the following behavioural analyses, RW indicates the reward window condition, coded as a fixed factor (0 for wide and 1 for narrow). TT represents the target time (2.25/3.5/4.75/6.0/7.25 s), also coded as a fixed factor. Ntrial is the trial number within the block.

***Outlier rejection:*** Trials in which waiting times deviated by more than 3 standard deviations from the pooled mean across all participants and conditions (0.24% of all trials) were discarded. Because |δWT| is computed from consecutive waiting times, the trial immediately following each outlier was also excluded to avoid contaminating |δWT| with the preceding outlier value.

***Statistical analysis:*** All statistical analyses were conducted in R (4.0.3). Linear mixed-effects models were fit with the lme4 package (Bates et al., 2014); Type II Wald χ² tests for fixed effects were obtained via the car package (Fox & Weisberg, 2020), with degrees of freedom calculated using the Kenward–Roger approximation where applicable (lmerTest) (Kuznetsova et al., 2017). Generalised linear mixed-effects models (binomial family) used the bobyqa optimiser (Powell, 2009). Post-hoc comparisons were computed using estimated marginal means (emmeans) (Lenth et al., 2019) with Tukey correction for multiple comparisons; trends were compared using lstrends from the lsmeans package. Bayesian analyses were performed using brms (Bürkner, 2017) with default weakly informative priors.

All models were fit using single trials from all subjects, and a random intercept term ([β_0,s_]) was added to account for between-subject variability (Baayen, 2008). We did not include random slopes in our principal models because more complex random effects structures failed to converge in our dataset. Parsimonious random effects structures have been shown to provide better-balanced control of Type I and Type II errors than maximal structures in datasets of this size (Matuschek et al., 2017).

To test whether participants’ trial-by-trial adjustment (|δWT|) was modulated by our manipulations of interest, |δWT| was entered as the dependent variable in a mixed-effects model analysis with trial number (1 to 10, for each block) as a fixed effect.

In this model (Eq. 1), |δWTi| indicates the absolute difference between the waiting time in trial *i* and the waiting time in previous trial *i-1*. This value was square-root transformed to better approximate a Normal distribution (Baayen, 2008).

$$\begin{array}{l} \sqrt{\left|\delta WT_{i}\right|}\text{​}\;\sim \;\beta_{0}\;+\beta_{1}RW_{i}+\beta_{2}TT_{i}+\beta_{3}Ntrial_{i}\\ \;+\beta_{4}RW_{i}*TT_{i}+\beta_{5}RW_{i}*Ntrial_{i}+\beta_{6}TT_{i}*Ntrial_{i}\\ \;+\beta_{7}RW_{i}*TT_{i}*Ntrial_{i}+\left[\beta_{0,s}\right]. \end{array}$$ (1)

We expected participants would use the signed feedback provided on a trial-by-trial basis to adjust their waiting times in a directional manner. Namely, we expected that they would wait longer in the next trial after receiving “too early” feedback, and that they would act faster after receiving “too late” feedback. To test whether this was the case, the signed δWT (not absolute) was also entered as the dependent variable in a mixed-effects model analysis. In this model (Eq. 2), δWT represents the signed change in waiting time, reflecting trial-by-trial adjustments based on feedback type. “sFeedback” indicates the signed feedback received in the previous trial (“too early,” “perfect,” “too late”), coded as a fixed factor. This model allows testing the hypothesis that the feedback direction predicts the magnitude and direction of adjustments in response times.

$$\begin{array}{l} \delta WT_{i}\sim \;\beta_{0}+\beta_{1}RW_{i}+\beta_{2}TT_{i}+\beta_{3}sFeedback_{i-1}+\beta_{4}RW_{i}*TT_{i}\\ \;+\beta_{5}RW_{i}*sFeedback_{i}+\beta_{6}TT_{i}*sFeedback_{i-1}\\ \;+\beta_{7}RW_{i}*TT_{i}*sFeedback_{i-1}+\left[\beta_{0,s}\right]. \end{array}$$ (2)

Next, to check how close participants got to the optimal acting time over multiple trials, and to investigate how the reward window condition affected that metric, we also computed, for each trial, the unsigned timing error between participants’ waiting time and block optimal waiting time (|Timing error|). In this model (Eq. 3), |Timing error| represents the absolute deviation in response times, which is square-root transformed to better approximate a Normal distribution (Baayen, 2008).

$$\begin{array}{l} \sqrt{\left|{\text{Timing error}}_{i}\right|}\text{​}\;\sim \;\beta_{0}+\beta_{1}RW_{i}+\beta_{2}TT_{i}+\beta_{3}Ntrial_{i}\\ \;+\beta_{4}RW_{i}*TT_{i}+\beta_{5}RW_{i}*Ntrial_{i}+\beta_{6}TT_{i}*Ntrial_{i}\\ \;+\beta_{7}RW_{i}*TT_{i}*Ntrial_{i}+\left[\beta_{0,s}\right]. \end{array}$$ (3)

Finally, to test how our experimental conditions affected the feedback probability, a logistic mixed‐effects model predicting feedback (0/1) from reward window, target time (2.25/3.5/4.75/6.0/7.25 s), and trial number (with random intercepts for ID) was conducted. In this model, the dependent variable is Feedback, coded as a binary outcome (0/1), where 0 included both “too early” and “too late” trials. A logistic link function is applied to model the probability of receiving positive feedback (Feedback = 1). A logistic mixed-effects model (binomial family) was applied, using the bobyqa optimiser to ensure convergence (Powell, 2009).

$$\begin{array}{l} logit\left(p\left(Feedback\right)\right)\;\sim \;\beta_{0}+\beta_{1}RW_{i}+\beta_{2}TT_{i}+\beta_{3}Ntrial_{i}\\ \;+\beta_{4}RW_{i}*TT_{i}+\beta_{5}RW_{i}*Ntrial_{i}+\beta_{6}TT_{i}*Ntrial_{i}\\ \;+\beta_{7}RW_{i}*TT_{i}*Ntrial_{i}+\left[\beta_{0,s}\right]. \end{array}$$ (4)

#### 2.5.2 EEG data analysis

***Preprocessing:*** EEG data were processed using Matlab R2021a (MathWorks) and EEGLAB version 2021.0 (Delorme & Makeig, 2004). Scalp and eye electrodes were re-referenced to the average of two mastoid electrodes. Continuous EEG and EOG data were filtered with a 0.01 Hz high-pass filter. These filters were applied off-line using a 5th order Butterworth filter with zero phase shift. Data were downsampled to 200 Hz and filtered with a 30 Hz low-pass filter. Data were epoched from −2 to 1 s around action for readiness potential analysis, and additionally also around [-1 to 2 s] trial start for beta power analysis.

Baseline correction was performed using a 10 ms window centred on the time of the action [–5 to +5 ms relative to keypress] (Khalighinejad et al., 2018). RP recordings are conventionally baseline corrected by subtracting the average signal value during an arbitrary-chosen window before the time of the action (e.g., 2.5 until 2 s before action). However, this involves the implicit but questionable assumption that motor preparatory processes occur in the final 2 s before action onset but not before (Verbaarschot et al., 2015). Our choice of baseline at the time of the action avoids making assumptions about how or when preparatory processes begin. This is important since our experimental design specifically manipulated the target time of action between 2.25 and 7.25 s, meaning that any pre-action baseline window risks falling within an already-active motor preparation period in long-target blocks. Although the 0.01 Hz high-pass filter applied to the data removes very-slow DC drifts and could in principle make baseline correction unnecessary, 0.01 Hz is a gentle cutoff, and substantial slow drift can survive. We, therefore, retained a minimal 10 ms baseline correction at the action timepoint as a conservative step. This approach is closer in spirit to the no-baseline-correction strategy advocated by Schurger (2018) than to conventional pre-action baselining, in that it anchors the reference at a theoretically meaningful, condition-invariant timepoint rather than at an arbitrary pre-action window.

Independent component analysis (ICA) was computed on the continuous data using the EEGLAB runica algorithm (Delorme et al., 2007). Vertical and horizontal eye movement components were identified by visual inspection and removed from the signal.

Finally, artefact rejection was performed by removing all epochs with >120 μV fluctuations from baseline in any of the preselected channels (Fz, FCz, Cz, FC1, FC2 for the RP (Travers et al., 2021), or contralateral C1 and C3 for motor beta analysis).

***RP slope extraction:*** To measure the slope of the readiness potential (RP) within each individual trial, the amplitude of the EEG signal was averaged across electrodes Fz, FCz, Cz, FC1, and FC2. For each trial, the slope of this average signal over the 2 s preceding action onset was estimated by fitting a linear regression directly to the raw samples (at 200 Hz, i.e., 401 samples per trial) of the pre-action window. This procedure was performed separately for each trial and each participant, yielding one slope value per trial. Trials in which the mean pre-action EEG amplitude deviated by more than 3 SD from the participant’s mean were excluded, along with the immediately subsequent trial.

***Time–frequency decomposition:*** To compute time–frequency decompositions of the single-trial activity, we used a Hanning taper (window length, 0.4 s; time steps, 0.05 s; frequency steps, 1 Hz; frequency smoothing, ±2.5 Hz) for the range 1–35 Hz using the FieldTrip toolbox (Oostenveld et al., 2011). We then averaged over the beta band range (13–30 Hz) and across the preselected contralateral electrodes (C1 and C3) encompassing central and contralateral motor areas to perform subsequent analysis.

***Statistical analysis:*** First, we aimed to investigate whether the slope of the readiness potential was related to our variables of interest.

Single-trial RP slope was modelled using a linear mixed-effects model with |δWT|, reward window, and waiting time as fixed effects (Eq. 5; see Statistical analysis). Convergence of the maximal random effects structure was attempted using both brms and blme (Chung et al., 2013); both failed, providing direct empirical support for the Matuschek et al. (2017) argument (see Supplementary Note 3 for details).

Following the same logic as the behavioural analyses, we used participants’ actual waiting time rather than target time to ensure a closer alignment between the EEG signals and the modelled behaviour.

$$\begin{array}{l} RP\;Slope_{i}\;\sim \;\beta_{0}+\beta_{1}|\delta WT{|}_{i}\;+\beta_{2}RW_{i}+\beta_{3}WT_{i}\\ \;+\beta_{4}\left|\delta WT{|}_{i}*RW_{i}+\beta_{5}\right|\delta WT{|}_{i}*WT_{i}\;+\beta_{6}RW_{i}*WT_{i}\\ \;+\beta_{7}|\delta WT{|}_{i}*RW_{i}*WT_{i}+\left[\beta_{0,s}\right], \end{array}$$ (5)

where RP slope was computed for each individual trial separately as described above, WTbin(i) indicates waiting time bins in quintiles, computed across blocks and conditions, RW(i) indicates the reward window condition for that block in a binary manner (0/1 for wide/narrow), and |δWT|(i) indicates the magnitude of trial-by-trial motor updates, computed as the absolute value of the difference WT(trial i) – WT(trial i−1). We used the absolute value of δWT to capture the magnitude of behavioural adjustment between trials, irrespective of whether participants acted earlier or later. Note that a more precise motor plan (Travers et al., 2021) allows timing updates in either direction.

Second, we aimed to test whether beta power over motor areas contralateral to the effector (right) hand varied with participants’ waiting times, the reward window condition, and the direction of trial-by-trial waiting time updates. Unlike for the RP analysis above, we did not have a strong a priori hypothesis as to when exactly effects might emerge. Thus, instead of taking a summary measure, we chose to run a time-resolved analysis that allowed us to remain agnostic about the effect timings, and rely on cluster-based permutation for statistical testing. Thus, we first ran the following single-trial regression for each point in time of the beta trajectory:

$$\begin{array}{l} Beta\;power_{i,t}\;\sim \;\beta_{0}+\beta_{1}\delta WT_{i}+\beta_{2}RW_{i}+\beta_{3}WT_{i}\\ \;+\beta_{4}\delta WT_{i}*RW_{i}+\beta_{5}\delta WT_{i}*WT_{i}+\beta_{6}RW_{i}*WT_{i}\\ \;+\beta_{7}\delta WT_{i}*RW_{i}*WT_{i}+\left[\beta_{0,s}\right], \end{array}$$ (6)

where Beta power is the beta power at electrodes C1 and C3, for times t:[–0.2 to 2 s] around trial onset for stimulus-locked traces, and [-2 to 0.5 s] for response-locked ones, in each trial. This model was fit to all participants individually, and both beta power and each regressor were z-scored before fitting.

Key to our hypotheses, positive β3 coefficients would indicate that trials with long waiting times were associated with higher beta power than trials where participants acted faster. Note that, in contrast to the RP analyses above, we use signed rather than absolute values of δWT(i). This is because, in line with the literature (Kelly et al., 2021; Steinemann et al., 2018), we hypothesise that higher or lower beta power at trial start reflects lower or higher levels of urgency, respectively. Therefore, the sign of the trial-by-trial updates might matter. Thus, positive β1 coefficients indicate that trials where participants act later than on the previous trial (positive δWT) are associated with higher contralateral motor beta power. All other main effects and interactions are included for consistency with RP analysis and for completion.

As in the RP analysis above, we decided to use waiting time instead of target time for our main analysis to ensure a closer alignment between the EEG signals and the modelled behaviour. However, for the sake of completion, we repeated the analysis in Eq. 6 using each block’s target times (TT) instead of waiting times.

To test whether our regression coefficients of interest were significant, we ran a two-tailed cluster-based permutation test using the FieldTrip toolbox (Maris & Oostenveld, 2007). We ran two tests using 10,000 permutations, 1 for each of our 2 time windows of interest: around trial onset, and around response.

#### 2.5.3 Computational modelling and simulations

Finally, we aimed to explore whether an extended version of the noisy accumulator model postulated by Schurger et al. (2012) as a computational mechanism underpinning the RP characteristics could recapitulate our observations. To this aim, we followed the standard accumulation model with the formulation in Eq. 7:

$$\delta y_{t}=\;\left(I-k*y_{t}\right)*\Delta dt+\;c\zeta_{t}*\sqrt{dt},$$ (7)

where δ*y_t_* indicates the change in the state of the accumulator *y* at each point in time *t, I* indicates a fixed input to the accumulator, *k* is leak (linear decay in *y*), ξ is Gaussian noise, and *c* is a noise scaling factor. In all our simulations, we used fixed values of leak (k = 0.05) and noise scaling (c = 0.1). Default input values were set to I = 0.175, but were modulated in different ways in our various simulations.

Note that in all simulations below, urgency and motor plan precision are treated as fixed parameters, with values chosen to span the range of conditions in our experiment. The simulations, therefore, model the within-trial accumulation process conditional on a given learned target time and precision level, rather than the across-trial learning dynamics by which those parameters are acquired.

First, we explored two ways in which urgency constraints (or how fast participants were required to act) could be implemented in the accumulator model. The urgency (U) parameter captured the differing time pressure to act in our various target time conditions, with lower values indicating the least urgency, and higher values the most urgency. In the first set of simulations (Fig. 5A–C), we allowed input strength to linearly scale with urgency by a factor *s* (I = s*U, where U = [-2,-1,0,1,2]) so that stronger input was provided in blocks where target times were shorter. Input was assumed to be constant over time. In the second set of simulations (Fig. 5D, E), we instead assumed urgency was implemented by modulating distance-to-bound for the accumulation in each target time condition, and we simulated that by adding a starting point bias *z* that linearly scaled with urgency by a factor *s* (z = s*U, where U = [1,2,3,4,5]). In these simulations, we assumed the input *I* to be the same across urgency conditions, and constant over time. The scaling factor *s* was set to s = 0.15 in all simulations, and urgency values were chosen arbitrarily to produce comparable waiting time distributions in our various simulations.

Finally, we explored whether dynamic modulations of input over time could account for increasing motor timing precision. In a third set of simulations (Fig. 5G–I), we changed input strength during trial time as follows:

$$I_{t}=I-s*Precision\;\;\;if\;t<\left(TW+0.5*\zeta \right)$$

$$I_{t}=I+s*Precision\;\;\;if\;t\geq \left(TW+0.5*\;\zeta \right),$$

where TW indicates the target waiting time (defined as the average waiting times obtained in the previous set of simulations), ξ is Gaussian noise to add some variability to the time at which input strength increased on a trial-by-trial basis, and Precision is a scalar value indicating either weak or strong input modulations (set to 1 or 5 in our simulations), linearly scaled by s (s = 0.15). Higher precision values result in inhibited default input I (I = 0.175) early in the trial, and produced a stronger boost close to the target time.

All data and analysis code associated with this study are openly available on the Open Science Framework (OSF) at: https://osf.io/26fm4/.

## 3 Results

### 3.1 Behavioural results

Participants (N = 25) learned the optimal time to make a simple action, based on feedback (Fig. 1A, C). The feedback depended on two distinct parameters, which varied independently across blocks: the target time at which participants should act, and the width of the window around the target where actions might be rewarded. Thus, when participants’ actions were likely to be rewarded in a wide window around target times (Fig. 1B), they could achieve high reward rates even if their plan of when to act was imprecise. Conversely, a narrow reward window required motor plans with greater timing precision to achieve the same reward rates.

We first verified that participants’ behaviour indeed varied across our experimental design factors.

The time of action was predictably earlier, and also more precise for shorter target times, recalling Weber’s Law (Gibbon, 1977). More interestingly, the time of action stabilised as the block progressed, as marked by a progressive narrowing of the action time distribution. This narrowing was predictably more marked in the narrow reward window condition than in the wide reward window condition (reward window × target time × trial interaction on waiting times: Χ²(4) = 21.15, p = 0.0003; Fig. 2F).

**Fig. 2.**
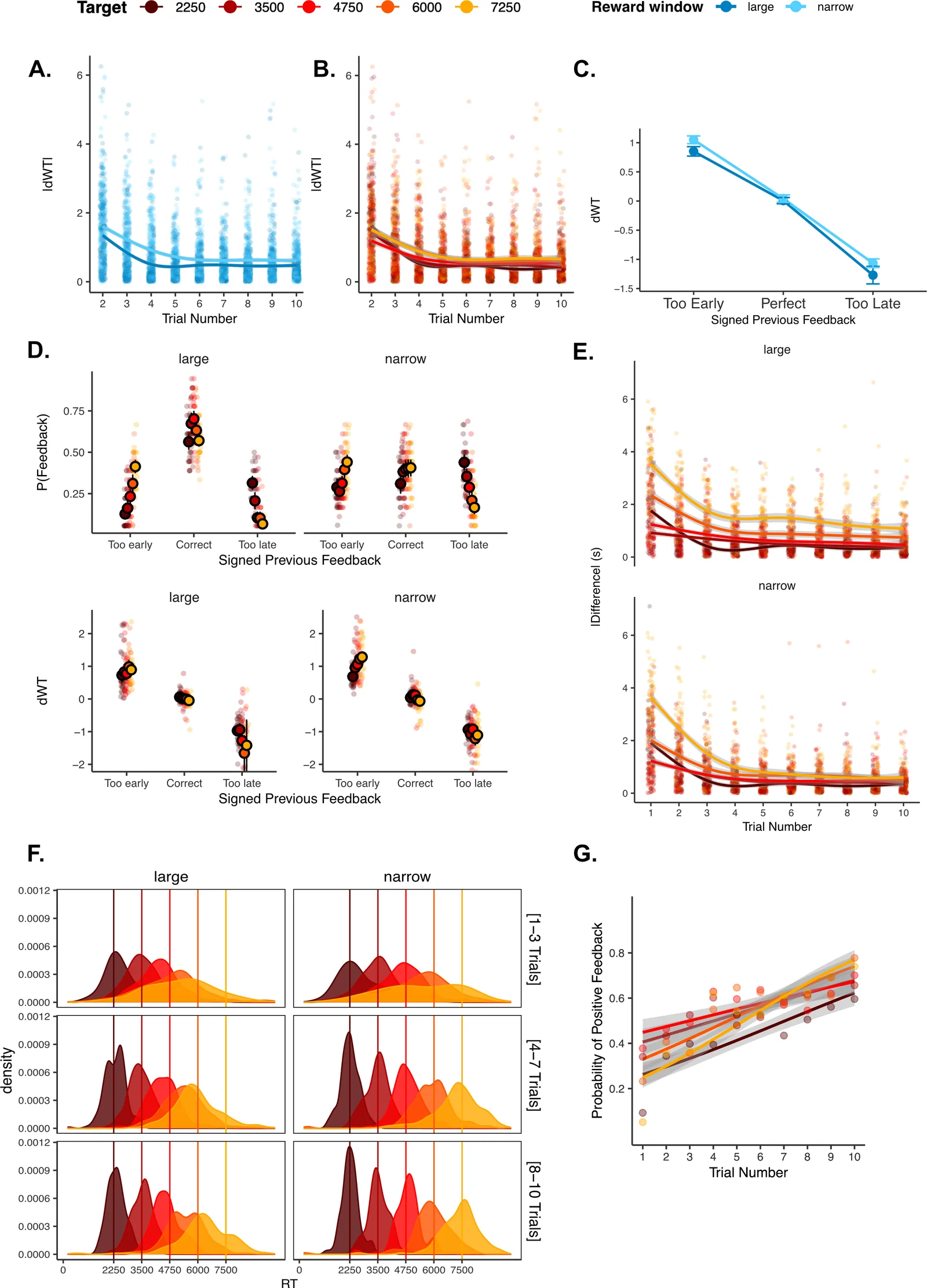
Behavioural Results. (A) Results of the analysis of |δWT|. Trial-by-trial updates in waiting time (|δWT|), during the 10 trials in a block, sorted by reward window conditions. Participants made overall smaller adjustments to their waiting times and reached a plateau performance faster in the wide reward window condition. Points show raw trial-level |dWT| values. Lines show GAM (Generalised Additive Model) smooth with 95% CI. (B) Trial-by-trial updates in waiting time (|δWT|), sorted by target time. The speed at which participants reached a stable waiting time was inversely related to target time, being fastest in shorter target time blocks. The average size of trial-by-trial |δWT| also scaled with target waiting time and was highest in longer target time blocks. Points show raw trial-level |dWT| values. Lines show GAM smooth with 95% CI. (C) Average change in waiting time (δWT) as a function of signed previous feedback, separated by reward window (wide vs. narrow). Adjustments in δWT follow the direction of the feedback: “Too early” feedback results in an increase in δWT, while “Too late” feedback leads to a decrease. The steepness of these adjustments suggests sharper corrections in the narrow reward window condition than the wide one, reflecting greater sensitivity to temporal errors when feedback constraints are tighter. Points show estimated marginal means (EMMs) from the fitted model; error bars indicate 95% confidence intervals. (D) Top panel: probability of receiving positive feedback as a function of signed previous feedback (“Too early”, “Perfect”, “Too late”), separated by reward window (wide vs. narrow) and target time. Faint points represent participant-level proportions of each feedback type; larger points indicate group means with bootstrapped 95% confidence intervals. Bottom panel: changes in waiting time (δWT) as a function of signed previous feedback, separated by reward window (wide vs. narrow) and target time. Too early or too late feedback resulted in compensatory adjustments in waiting time, with sharper adjustments observed in the narrow reward window condition, reflecting tighter feedback constraints. Points show raw trial-level |Difference| values. Lines represent GAM smooths with 95% confidence intervals. (E) Results of the analysis of |Timing error|. Absolute average size of trial-by-trial difference between participants’ waiting times and target times (|Timing error|), sorted by target time and split by reward window. The progressive decrease in the average size of trial-by-trial |Timing error| with Trial number was greater in the narrow than in the wide reward window condition. The average size of trial-by-trial |Timing error| also scaled with target waiting time and was greater in longer target time blocks. (F) Waiting time trial-by-trial evolution of waiting times for each combination of reward window and target time. Shorter target times tended to elicit faster waiting times, while longer target times showed delayed responses. The narrow reward window condition generally produced a steeper stabilisation in waiting times over trials compared with the wide reward window condition. (G) Feedback probability over trials. Trial-by-trial evolution of the probability of receiving positive feedback. Blocks with longer target times exhibited slower increases in positive feedback probability, suggesting a more gradual adaptation to the optimal waiting time compared with blocks with shorter target times. Lines show fitted probabilities from a binomial GLM (95% CIs). Points show empirical proportions of positive feedback at each trial. Unless otherwise stated, error bars indicate SEM.

The probability of positive feedback, indicating successful performance on the action-timing task, unsurprisingly evolved across trials, indicating learning. Performance improvement was slower in blocks with longer target times (target time × trial interaction effect on p(positive feedback): Χ²(4) = 23.47, p = 0.0001; Fig. 2G). More interestingly, this effect interacted with the reward window such that the narrow reward window condition yielded lower positive feedback probabilities in blocks with shorter target times (reward window × target time interaction on feedback probability: Χ²(4) = 17.77, p = 0.001; Fig. 2D, top panel). Further details are given in Supplementary Note 1.

To capture how participants adjusted their behaviour during learning, we examined three complementary measures of how action timing adapted across trials: the unsigned timing error relative to the target time (|waiting time – target time|), the absolute change in waiting time from one trial to the next (|δWT|), and the signed change in waiting time based on prior feedback (δWT).

As participants learned, the unsigned timing error between their waiting times and the target times (|Timing error|) decreased progressively. This decrease was faster and more pronounced in the narrow reward window condition, and it exhibited the same dynamics but higher error magnitudes in longer target times (reward window × target time × trial interaction on |timing error|: Χ²(4) = 16.56, p = 0.002; Fig. 2E).

We reasoned that participants would use feedback to learn the optimal time to act. Thus, the trial-by-trial change in waiting time before action (|δWT|) provides a potential marker of participants’ updating a representation of the correct time to act. When the target time is unknown (i.e., at the beginning of the block), building an internal representation of the optimal waiting time may require exploring a wide range of waiting times, foraging in search of informative feedback. Conversely, later in the block, as the optimal waiting time is learned, participants should update the time of acting only slightly between one trial and the next, as they acquire certainty about the correct time to act, and shift from exploration to exploitation, Travers et al. (2021). Further, we anticipated stronger updating, and thus greater |δWT|, in the narrow reward window condition, where finer temporal adjustments are required to align with the optimal waiting time.

The trial‐by‐trial analysis of absolute waiting time adjustments (|δWT|) predictably showed that participants modified waiting times less in the wide reward window condition compared with the narrow reward window condition (reward window × trial interaction on |δWT|: Χ²(1) = 13.56, p = 0.0002; Fig. 2A). Further, the time at which |δWT| plateaued was inversely related to target time: blocks with shorter target times quickly achieved a stable waiting time, whereas blocks with longer target times not only took longer to stabilise but also showed wider average adjustments (target time × trial interaction on |δWT|: Χ²(4) = 22.06, p = 0.0002; Fig. 2B). This pattern may, at least in part, reflect Weber-like scaling, where temporal variability increases proportionally with the mean interval duration.

Crucially, the stabilisation of signed waiting time adjustments (δWT) closely followed the sign of the received feedback (target time x signed previous feedback interaction on δWT: Χ²(8) = 81.99, p < 0.0001). When the feedback indicated “too early,” participants waited longer in the next trial (positive δWT), while “too late” feedback led to shorter response times (negative δWT) in the following trial (Fig. 2C, D). In the narrow reward window condition, once participants learned when was the right time to act, they kept making “back-and-forth” adjustments towards the target time in order to hit the target *precisely* at the time when rewards were most likely (Fig. 2D). This stands in contrast with the wide reward window condition. In those blocks, participants quickly identified a waiting time that was occasionally rewarded, and took it as a starting point to anchor their waiting times. Thus, while in the narrow reward window condition participants developed a very precise internal model of *when* they should act and strived to execute their actions at that exact time, in the wide reward window condition their model was less precise, and remained less precise than the narrow reward window for the whole duration of the block (reward window X signed previous feedback interaction on δWT (Χ²(2) = 12.36, p = 0.0021).

Together, these findings demonstrate that while the wide reward window condition makes it easier to obtain positive feedback, it hinders the development of a motor plan based on precise representations of a specific time to act. In contrast, the narrow reward window condition, although associated with a higher error rate, drives greater trial-by-trial adjustments that ultimately support the acquisition of optimal action timing.

### 3.2 The readiness potential reflects the precision of motor timing plans

The RP amplitudes are predicted to be higher when individuals have a precise estimate of the correct time to act, as a result of learning from feedback (Travers et al., 2021). RP amplitude is measured relative to a baseline period, but defining the baseline period in the context of self-paced actions is problematic (see above). For this reason, the RP *slope* has been proposed as a more relevant index of motor preparation (Schurger, 2018). We, therefore, extracted RP slope over a 2 s period preceding action, and found no main effect of waiting time on RP slope (Eq. 5; Waiting time (χ²(1) = 2.15, p = 0.14) Fig. 3A, top).

**Fig. 3.**
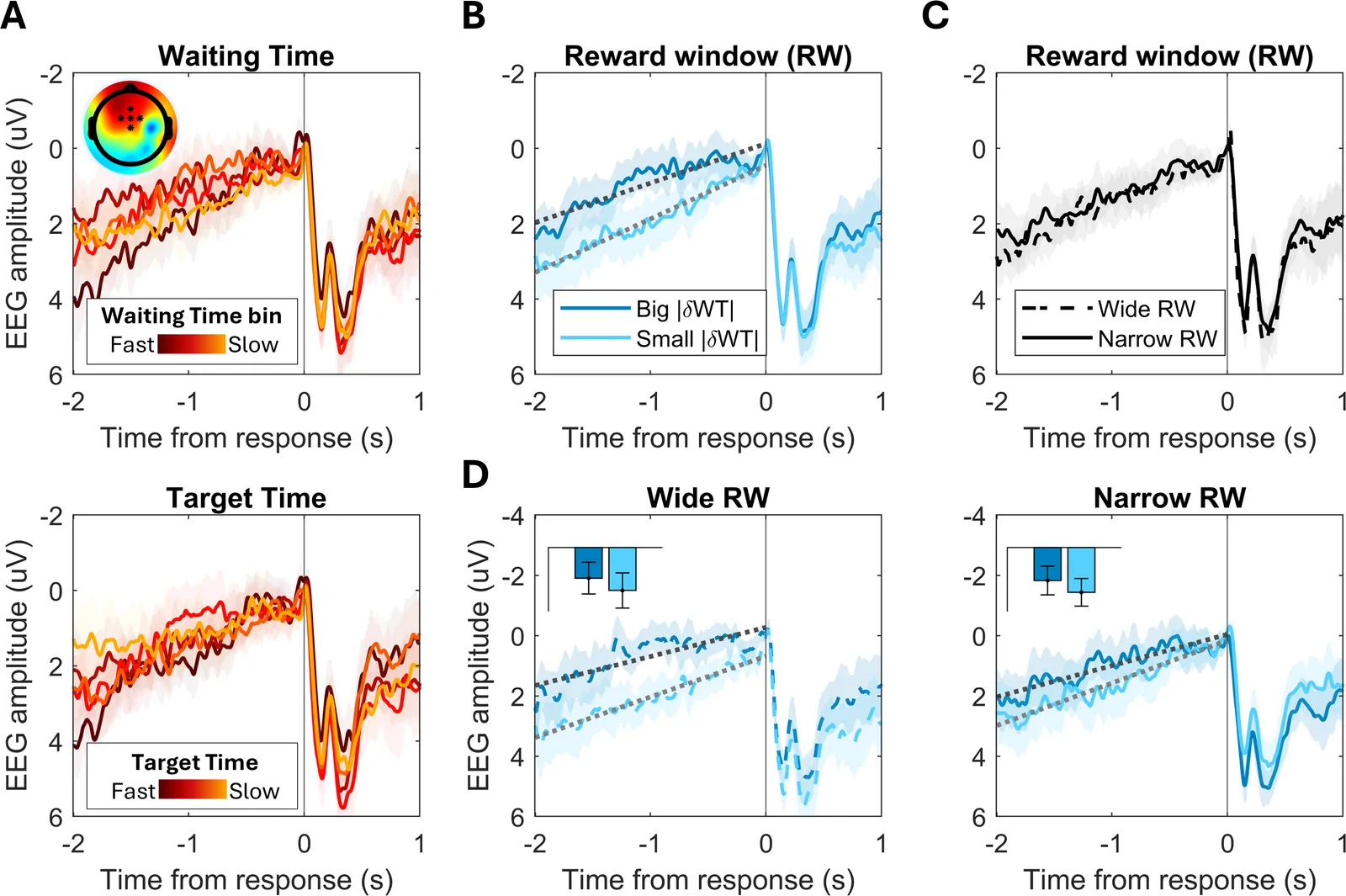
(A) Grand-averaged readiness potential (RP) amplitude (mean ± SEM, averaged over electrodes Fz, FCz, Cz, FC1 and FC2). Data are aligned to the time of action and grouped in five waiting time bins (*top*) or sorted by target times (*bottom*). Topographies indicate the average amplitude across all conditions between [-2 and –1.5 s] with respect to action, where the deviation from 0 is maximal given our baselining at the time of action. (B) RP amplitude (mean ± SEM), plotted after median splitting data in big and small |δWT| trials, averaging across reward and target time conditions. This data sorting effectively captures learning stage, where big |δWT| trials correspond to the initial, exploratory trials (before learning), and small |δWT| trials correspond to later stages in each block, where participants had already learned when to act. (C) RP amplitude (mean ± SEM) sorted by feedback conditions. (D) RP amplitude (mean ± SEM) sorted by learning stage and reward condition, illustrating the interaction between these two factors. The slope of the readiness potential became significantly steeper with learning, but only significantly so in the narrow reward window condition. Bar graph insets indicate average slopes (mean ± SEM) for each condition, computed based on the average traces for each participant.

Next, we investigated how RP slope changed with learning. We used the update in waiting time (|δWT|) as a trial-wise measure of learning. Participants transition from an exploration phase where they do not know the right time to act, and, therefore, make multiple large adjustments, to an exploitation phase, where small adjustments to action timing are indicated by frequent rewards for a given waiting time (see Fig. 2A, B). However, as discussed above, a small absolute update may be associated with motor time representations of differing precision in our two reward window conditions. In the narrow reward window condition, small updates indicate precise fine tuning of action execution processes around a well-learned internal model. In the wide reward window, small updates might instead reflect stochastic fluctuations. Since these did not affect reward, they would, therefore, be irrelevant to future actions. This distinction suggests that the same magnitude of behavioural change can carry different psychological meanings depending on the task environment.

The RP slope analysis revealed steeper slopes once participants had learned the correct timing and were making only minimal trial-to-trial updates to waiting time. This increase in RP slope with learning was more marked in the narrow reward window condition, reflecting the precision of the learned motor plan (Fig. 3B), as shown by a significant |δWT| by reward window interaction (χ²(1) = 4.31, p = 0.038; Fig. 3D, right). The analogous effect was not significant in the wide reward window condition, perhaps because small |δWT| updates were not linked to a precise representation of desired target time, and thus to a precise motor timing plan (Fig. 3D, left). To assess the robustness of these findings, we additionally re-fit Eq. 5 within a Bayesian framework (brms) and using classical per-subject regressions tested at the group level with one-sample t-tests (see full details on Supplementary Note 3). No main effect of reward window condition on RP slopes was found (χ²(1) = 0.44, p = 0.51). See Supplementary Note 2 for full statistical details of this analysis.

### 3.3 Contralateral motor beta power at trial start scales with waiting times

We also observed effects consistent with target motor time on beta power over contralateral motor cortex. Participants always responded using the right hand, and we, therefore, focused on left hemisphere electrodes [C1 and C3]. Contralateral beta power has been shown to always reach systematic threshold values at the time of action, regardless of action latency and other task constraints (Kelly et al., 2021; McCone, 2024; McCone et al., 2024; O’Connell et al., 2012), consistent with it reflecting an action threshold. However, waiting time and urgency manipulations have been shown to result in changes in the excursion observed in beta-band contralateral power, with bigger differences between beta-power at trial start and at action time in slow, low-urgency conditions compared with fast, high-urgency ones. On this account, the action-triggering bound itself is fixed, and is indexed by a stereotyped low value of contralateral beta power, but the starting point of motor preparation, indexed by beta power at trial onset, can be strategically adjusted. Setting this starting point closer to or further from the fixed bound shortens or lengthens, respectively, the distance the accumulation process must travel before action is triggered. The magnitude of the drop in beta power between trial start and action thus reflects this strategic adjustment of distance-to-bound rather than any change in the bound itself.

We did observe systematic changes related to waiting time in initial beta power according to the subsequent action latency (p = 0.026, two-tailed cluster-based permutation test). Contralateral beta power at trial start was higher in blocks with longer target times than in those with shorter target times (Fig. 4A, *top*). Similar qualitative results were obtained when grouping data by target time rather than actual waiting time, although the cluster failed to reach statistical significance (Fig. 4A, *bottom*, p = 0.15, two-tailed cluster-based permutation test). Note that our analyses tested for the presence of linear scaling of beta power with target times, an effect apparent in the continuous grading observed across conditions. An extended analysis revealed that these urgency-related modulations were also observable over the ipsilateral motor cortex (Supplementary Fig. S1), but did not extend to the alpha range (8–12 Hz; see Supplementary Figs. S2 and S3). Contralateral beta power was not significantly modulated by the reward window condition (Fig. 4B, *top*). We further investigated whether the direction of motor updates (operationalised as δWT) influenced beta power. Note that, in contrast to the RP analyses above, we used signed rather than absolute values of δWT because beta power is hypothesised to increase/decrease with lower/higher urgency, respectively. No significant effects were found (Fig. 4B, *bottom*). Cluster-based permutation testing returned no significant clusters for any other interaction in Eq. 6. Of particular note for the present account, the waiting time × reward window interaction was non-significant at both trial onset and around the response, indicating that the scaling of contralateral beta power with required waiting time was comparable in narrow and wide reward window conditions. This is consistent with our interpretation that beta-power modulations reflect an urgency-related mechanism that operates independently of motor plan precision.

**Fig. 4.**
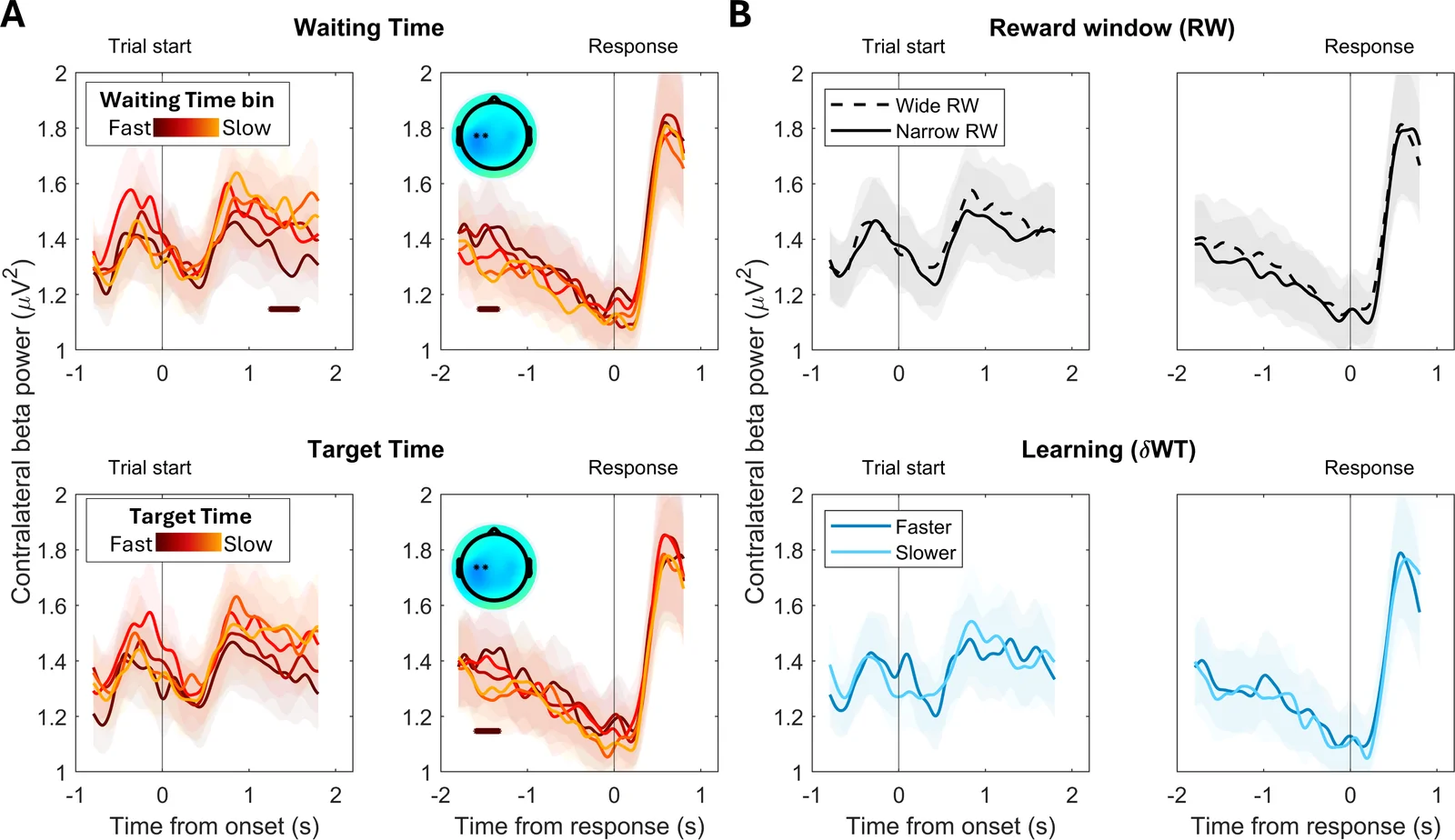
(A) Grand-averaged contralateral motor beta power (mean ± SEM; electrodes C1 and C3). Data are aligned to trial start (*left*) or response (*right*), sorted by binned waiting times (*top*) target times (*bottom*), averaged across reward window conditions. Motor beta power at the start of the waiting period scaled with the target time in each block, being higher the longer the waiting time. However, it reached comparable amplitudes at the time of action. Horizontal bars along the bottom indicate the times where the estimated effect of waiting time on motor beta power was estimated to significantly differ from zero (see Eq. 6). Topographies indicate the change in beta power (dB) between the 500 ms preceding trial start until the 500 ms preceding action, across all conditions. (B) Contralateral motor beta power (mean ± SEM; electrodes C1 and C3) illustrating that no main effects of reward window condition (*top*) nor motor update direction (δWT, *bottom*) were observed at the beginning of trials nor before action execution. For illustration purposes, we plot the top and bottom quintiles of trials sorted by dWT, which include trials where participants acted faster or slower than in the previous trial, respectively.

### 3.4 Neurally informed simulations recapitulate key observed results

Finally, we sought to investigate whether and how the observed behavioural and neural effects could emerge from a noisy accumulation process. To this aim, we built on the stochastic accumulator account of RP (Schurger et al., 2012). This model has been successfully used to predict self-paced waiting times, and can capture the ramping shape of the RP (Schurger et al., 2012). The original version of this model assumed a fixed level of urgency, operationalised as a fixed input to the accumulation process that is constant over time (Schurger et al., 2012). This “input” can be interpreted as the drift rate in the context of this model and determines how fast the decision signal builds up, and, therefore, how quickly actions are triggered. The assumption that input is fixed and does not change across trials is reasonable in contexts where no constraints are placed on self-paced action timing, such as classic spontaneous action tasks (Libet et al., 1983). In our task, however, the time pressure to act (i.e., urgency) depended on a target time that was varied across blocks. Here, our aim was to investigate how urgency manipulations could be implemented in this stochastic model in a way that captured our empirical behaviour and EEG observations.

One way to modulate average waiting times involves changing the input to this accumulator (Schurger, 2018). Thus, we first tested whether allowing the time-invariant input to linearly vary with target times could account for our observed behavioural and neural data (Fig. 5A). While changing the strength of the input to the accumulator model can produce differences in waiting time distributions akin to the ones observed, it also predicts strong differences in the shape of the RP (Fig. 5A–C), which we did not find in our data (c.f. Fig. 3A). We, therefore, explored alternative ways in which the stochastic accumulator framework could account for our observations.

**Fig. 5.**
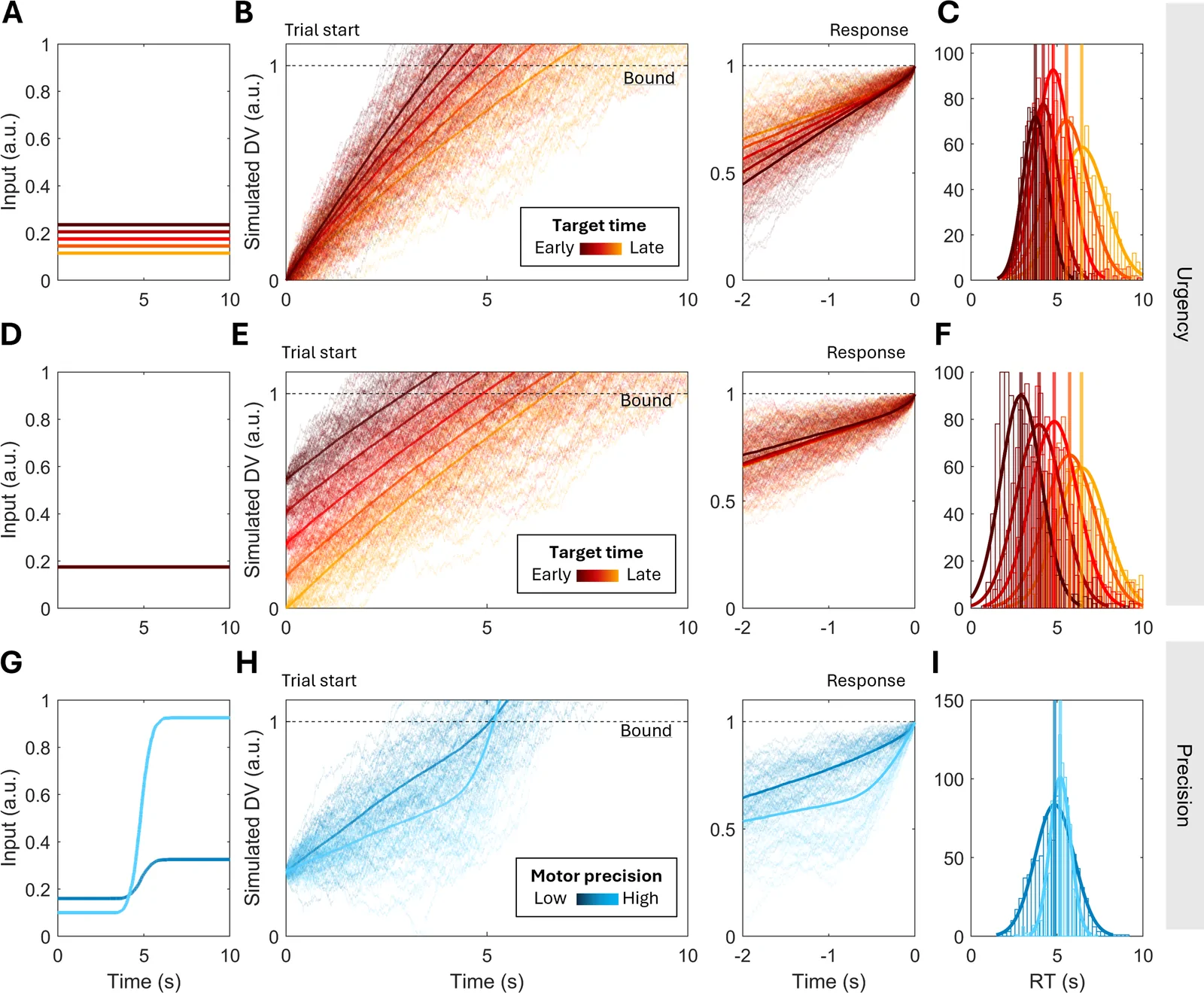
Simulations based on three variants of the stochastic accumulation model (N = 1,000). Top panels (A–D) model the effect of general urgency (or target times) as either a linear change in input strength (A–C) or as shift in the onset time of the accumulation process (D–F). Bottom panels (G–I) model the effect of motor plan (im)precision as a step change in input strength around the target time of action, showing only the intermediate urgency condition for illustration purposes. (A, D, G) Input strength during trial time. In (A), input was set to linearly scale with urgency constraints, but remained constant throughout trial time. In (D), input was also set to be constant throughout trial time, but it did not scale with urgency constraints (all conditions appear as flat, overlapping lines). In (G), input was modulated as a function of target time rather than being constant. Input was set to be lower before reaching a target action window, and increased around target time. The strength of this modulation is intended to capture the precision of a motor plan: the stronger the modulation, the more likely it is that actions will be triggered exactly at the intended time. (B, E, H) Simulated decision variables for various degrees of urgency (B, E) and motor precision (H), aligned to trial onset (*left*) or waiting time (*right*). Accumulation is assumed to start at time trial start (t = 0). Thin lines illustrate N = 50 sample trials, and thick lines illustrate the average across all N = 1,000 simulations. The action-aligned traces (right) effectively simulate the RP. RP slope scaled with trial time only when varying waiting time distributions were achieved by varying accumulator input (B), but not when they were achieved by means of a baseline shift (E). Further, RPs were steeper in conditions where input strength was strongly boosted close to target time, simulating a precise motor timing plan (E). The low and high motor precision conditions mirror the learning-related effects observed in our narrow band-width condition. (C, F, I) Waiting time distributions for various urgency levels (C, F), and for different motor plan precisions (F).

Another way to achieve systematically different waiting times involves changing the distance-to-bound for the accumulation process. The longer the distance-to-bound, the slower the average waiting times. Our finding that motor beta power varied as a function of waiting time at trial start, yet consistently reached a low and stable value just prior to action (Fig. 4A) clarifies how strategic adjustment of average waiting time might work. In essence, the level of motor activation at the beginning of the trial is under strategic control, but the actual trigger for action (i.e., the minimal beta-power at the time of action) is fixed. Thus, if a participant can set the starting beta power higher or lower, their mean waiting time will be longer or shorter, respectively.

To explore this new concept of strategic control over initial motor activation, the next set of simulations assumed a constant input (i.e., drift rate) to the accumulator (Fig. 5D) and a fixed decision bound, but introduced a baseline shift in the accumulation process that linearly scaled with target time (Fig. 5E). This modulation means that the stochastic decision signal began closer to threshold when actions needed to be triggered faster. The shorter distance-to-bound in fast action trials thus mirrors the empirical observation that beta power is lower at trial start in blocks with short target times and, given the constant power at action time, exhibits a lower excursion in those trials than in slow ones. This simulation produced plausible waiting time distributions and did not predict strong differences in RP slope for various waiting times (Fig. 5D–F). These simulations thus qualitatively recapitulate our empirical observations, which found no strong differences in RP slope for various waiting times (c.f. Fig. 3A).

Next, we sought to capture how modulating the precision of motor timing plan could reproduce the observed RP effects. The original noisy accumulator model assumes that, given a fixed level of urgency, the exact time of action primarily depends on noise. Here, we simulated a scenario where the input to the accumulator (or drift rate) was set to act as a continuous version of a trigger-like, internal go signal, with weaker strength earlier in the trial and a step-change close to target times, as in Travers et al. (2021). This model captured finer modulations of waiting times, for any given level of urgency (i.e., target times, in our task). Even when waiting times were on average the same, stronger input modulations led to smaller trial-by-trial waiting time updates (i.e., smaller |δWT|, leading to narrower waiting time distributions) than conditions with weaker input modulations. These simulations also recapitulated the RP slope effects observed in the narrow reward window condition (Fig. 5G–I), where steeper pre-action build-ups were observed after participants learned when the correct time to act was (c.f. Fig. 3B, D). In this implementation of the model, time-varying input modulations result in a two-stage accumulation process: a slow early accumulation (weak input signal or drift rate), and a fast, trigger-like one around the time of action (strong input signal or drift rate). In turn, the more precise the action time representations, the sharper the difference between these two phases is (Fig. 5G–I).

In sum, these simulations illustrate how two distinct mechanisms could explain the observed behavioural and neural data. First, general urgency modulations are consistent with a strategic adjustment of the distance to bound that the accumulation process needs to cover to trigger an action (Fig. 5D, E), with starting point shifts mirroring the observed empirical contralateral beta power results at trial onset (c.f. Fig. 4A). Second, the development of a temporally precise motor plan may involve changing the strength and temporal profile of the input to the accumulator (Fig. 5G–I), so that a strong, temporally precise internal go signal reduces the impact of noise in determining when exactly the accumulator will reach a bound, and resulting in smaller trial-by-trial-updates. Note that the goal of this exercise was not to identify the computational model that best captures our behavioural and neural effects, but rather to illustrate, as a proof of concept, one way in which the qualitative data patterns might emerge from distinct computational processes. Exploring alternative models and evaluating the best-fitting ones is beyond the scope of this paper.

## 4 Discussion

Learning to act just at the right time in any situation is an essential aspect of voluntary control. However, little is known about how prominent motor preparation signals in the human brain are involved in such a process. In this study, we used a task where participants learned the appropriate time to act in each block based on trial-by-trial feedback. We found that motor beta desynchronisation and the RP exhibit distinct changes as people learn when to act, suggesting that they play different functional roles: while motor beta power modulations are consistent with a general urgency regulation mechanism associated with how fast or slow an action should be, the steepness of the RP, in contrast, may reflect neural processes involved in fine tuning the exact timing of action. Our extension of a stochastic accumulator model can recapitulate these observations and suggests that precise time interval production may be achieved through a combination of two synergic mechanisms: a flexible modulation of the starting point for accumulation and temporally precise changes in the input (i.e., drift rate) to an internal action-triggering accumulator signal.

Our use of different target times across blocks effectively manipulated the urgency to act in each condition. In turn, our two different reward schemes affected how people learned: in the wide reward window condition, participants stopped making big adjustments to their waiting times (|δWT|) relatively early on within blocks (Fig. 2A), because receiving positive feedback was possible in a broad range of time windows and they identified an occasionally rewarded waiting time within the first few trials (Fig. 2D, top panel). However, this also meant that they remained relatively far from the target times where rewards were most likely in each block (Fig. 2E). Because the reward window was wide, they had no need to develop a very temporally precise timing plan in order to receive positive feedback. After identifying a response window that was occasionally rewarded, participants remained within that time range, and made only small trial-by-trial updates (|δWT|) that slowly brought them closer to the target time (Fig. 2A). In contrast, in the narrow reward window condition, participants could only get positive feedback if they moved very close to the actual target time. Because negative feedback was directional (i.e., “too early”/”too late”), they developed a precise internal model of when they should act very early on. However, because rewards were likely only for a very restricted period of time, participants often received negative feedback, and they kept updating their trial-by-trial waiting times back and forth to try to act during the very narrow window where positive feedback was most likely (Fig. 2A). Thus, our two reward schemes differed in the precision of participants’ internal models about the right time to act, with small trial-by-trial adjustments (|δWT|) being associated with higher motor plan precision in the narrow than in the wide reward window condition. How were these evolving motor plans implemented in the brain? To answer this question, we turned to two prominent motor preparation signals known to play a key role in self-initiated action triggering: the readiness potential (RP) and motor beta desynchronisation.

We found that the shape of the RP was linked to how precise participants’ motor timing plans were (Fig. 3B), but not to the actual time of action (Fig. 3A). Following the literature, we used trial-by-trial waiting time updates |δWT| as a proxy for learning (Travers et al., 2021). We found that RP build-up was steeper when trial-by-trial waiting time updates |δWT| were small, but only significantly so in the narrow reward window condition (Fig. 3D) which is, as noted above, the one where small |δWT| values were more strongly linked to precise motor timing plans. Thus, our results are in line with previous research (Travers et al., 2021) showing increased amplitudes for the RP in trials with small waiting time adjustments. On this view, steeper RP slopes reflect a precise representation about exactly when to act. Importantly, the slope of the RP did not significantly vary with waiting times in our task (Fig. 3A). The lack of strong differences in RP slope across target times could not be explained by the original accumulator model of the RP (Schurger et al., 2012). However, a simple extension in which urgency is implemented as a shift in the starting point of accumulation rather than as a change in the strength of time-invariant input does accommodate our data. If the average RP reflects only accumulation of noise to an action bound, then an earlier action can only be achieved by stronger input, leading to more rapid accumulation—assuming the noise generator itself remains constant. This model, therefore, predicts steeper RP slopes for faster actions (Schurger, 2018; Trovò et al., 2021).

In addition, we found that RP shape varies systematically with learning in our narrow reward window condition. These findings are predicted if the strength of the accumulator input is modulated over time, with maximal strength around the target action time, and stronger modulations where the precision of motor plans is highest (see Travers et al., 2021), producing steeper slopes. While neurofeedback studies suggest that the RP is not under direct voluntary control (Schultze-Kraft et al., 2021), our finding that its shape is modulated by the precision of action plans suggests that it might nonetheless be sensitive to higher-order control processes.

We then turned to motor beta power. Consistent with the literature (Kelly et al., 2021; McCone, 2024; McCone et al., 2024), we found contralateral motor beta power at the time of action to be invariant across various waiting times (Fig. 4A, *right*). In contrast, beta power at the start of the waiting period scaled with each block’s target waiting times shortly after trial onset (Fig. 4A, left). Together, these two observations suggest a two-part role for contralateral motor beta power. First, the stereotyped low value of beta power at action time corresponds to a fixed action-triggering bound. Second, the level of beta power at trial onset corresponds to the starting state of the motor preparation process, a starting state that can be strategically adjusted closer to or further from the fixed bound to produce shorter or longer waiting times, respectively. The empirical signature of this account is precisely the combination we observe: an invariant beta value at action time and a target time-dependent beta value at trial onset. While previous work had identified contextual shifts in baseline beta power in blocked designs (Steinemann et al., 2018) and cued tasks (Kelly et al., 2021), this is the first task to show how these adjustments gradually evolve as a function of learning. Motor beta power and its underlying beta bursts have been linked to various aspects of movement, including inhibition in stop tasks (Enz et al., 2021), movement onset time (Little et al., 2019), vigour (Uehara et al., 2023), and motor control flexibility (Pierrieau et al., 2025), highlighting the critical role of this signal on motor control. Our findings add to this literature and suggest beta power modulation may form part of a flexible mechanism for action timing that is under voluntary control. Similar adjustments over motor beta power have been observed on a trial-by-trial level in recent interval reproduction tasks (McCone et al., 2024), where participants are required to replicate a time interval after listening to a sample cue.

Put together, our findings suggest that the RP and motor beta power may track two different aspects of the timing of action, with the former associated with precise temporal control of action execution and the latter with coarser, urgency-related modulation. Contralateral beta power desynchronisation is a ubiquitous feature of action planning, present in self-paced (e.g., Parés-Pujolràs et al., 2023; Gavenas et al., 2024; Little et al., 2019) and cued actions (Tzagarakis et al., 2010), as well as in evidence-informed perceptual decisions (Steinemann et al., 2018; Donner et al., 2009). Thus, we believe the urgency-control role we propose for it here is likely to extend to multiple contexts. However, the extent to which a central readiness potential is to be expected across tasks remains debated (see Khalighinejad et al., 2018; Schurger et al., 2021 for a review). Thus, whether our findings on its indexing of motor plan precision generalise to contexts other than self-paced tasks remains to be investigated.

Previous work has suggested that distinct neural circuitry may be associated with coarse (supra-second) and fine (sub-second) action timing control, with basal ganglia and the cerebellum as key respective candidate areas (Kunimatsu et al., 2018). The possibility of separate mechanisms involved in coarse and fine action timing control makes it plausible that distinct EEG correlates of such control processes can be identified, and it can also inform modelling efforts. The influential stochastic accumulator model, which successfully explains how the RP may emerge from a noisy accumulation process (Schurger et al., 2012), originally assumed a fixed level of urgency, which was operationalised as a fixed input or drift rate to the accumulation process. This assumption is reasonable in contexts where no constraints are placed on self-paced action timing, such as classic spontaneous action tasks (Libet et al., 1983; Parés-Pujolràs et al., 2019; Parés-Pujolràs et al., 2023; Schurger, 2018; Schurger et al., 2012). However, paradigms where different paces of action are required in different blocks effectively manipulate action urgency, so that it can no longer be assumed to be fixed across conditions. While changing the input to the accumulator would effectively result in various waiting time distributions, such a manipulation would also result in systematic changes in the shape of the RP across conditions, with steeper slopes predicted for stronger urgency conditions (Fig. 5A–C). However, we did not observe systematic differences in RP slope across our various target time conditions. Thus, we explored whether a simple extension of the model could account for our results. Based on the observation that motor beta power reached stereotyped levels at the time of action but was linearly modulated by target times at trial onset, we set a fixed bound for our accumulator but introduced a linear bias to starting points of the accumulation to bring decision variables closer to bound in blocks where actions needed to be fast. This recapitulated our observations that the shape of the RP did not vary across target time conditions, while achieving linearly scaled waiting time distributions.

Our account proposes that motor plan precision corresponds to the strength and temporal sharpness of an input delivered to the noisy accumulator that generates the RP. A precise plan (after learning, small |δWT| in our task) delivers a strong, temporally localised boost in input around the learned target time; an imprecise plan (before learning, big |δWT|) is associated with a weaker diffuse modulation. Two consequences follow for the action-locked average. First, individual trials rise more steeply towards the bound under high precision, because the late input boost is stronger and provides a larger deterministic push towards threshold. Second, the timing of bound crossing varies less across trials under high precision, because a more localised input leaves less room for noise to determine when the bound is reached. Both effects compound: the action-locked average is steeper not only because within-trial dynamics near action are steeper, but also because reduced cross-trial variability means averaging preserves more of that steepness rather than smearing it out. Conversely, a shallower RP slope under low motor plan precision reflects a combination of weaker within-trial input and greater trial-to-trial variability in the timing of bound crossing.

Our modelling, therefore, assumes two distinct phases within the accumulation process leading to actions. A first phase where accumulation proceeds at a very slow rate (weak input or drift rate), and a second one afterwards when a trigger-like event (strong input or drift rate) speeds up the race to bound. However, whether the brain in fact implements temporal estimation and action triggering in a single integrated process or as two distinct functions is something our data cannot disentangle, and further research will be needed to elucidate these questions.

Next, we modelled whether dynamic changes in input strength throughout the trial could account for the increase of RP slope with learning. The original implementation of the noisy accumulator model assumes that, given a constant level of urgency or time pressure to act, the exact time of actions primarily depends on noise. This is because the input to the accumulator is kept constant over time. However, such a model is not consistent with the fact that RP slopes systematically increased with learning, irrespective of mean action times. Thus, we aimed to explore whether allowing the input to the accumulator to change over time in a manner that depends on learned target action times could account for the observed RP results. Here, we modelled timing precision as a step change in input strength close to action target times, following previous suggestions (Travers et al., 2021). Our model assumes that the input to the accumulator is modulated by the participants’ representation of the target action time, such that the accumulator only receives weak input when it is too early to act, and instead receives a strong, trigger-like input closer to target action times. Varying the strength of this temporally precise boost in input close to the target time recapitulated the effects on RP slope associated with precise motor timing observed in our narrow reward window condition as well as in previous work (Travers et al., 2021).

In sum, our modelling of waiting times serves to illustrate some ways in which a stochastic accumulation model for the RP can be adapted to account for observations in a context with varying urgency constraints. Here, we built on the original stochastic accumulator model (Schurger et al., 2012) to test whether modifying it to include strategic, context-based modulations would allow the model to account not only for the spontaneous, “capricious” actions it was initially built to explain, but also for precisely controlled ones. The original model was designed for the classical spontaneous voluntary movement paradigm, where there is no target time and the imperative to act—modelled as the input to the accumulator—is deliberately weak and time invariant. Our task differs fundamentally, requiring participants both to learn an optimal time to act from feedback and to produce a temporal interval approximating that learned target. We, therefore, view the present modelling exercise as an attempt to extend the explanatory scope of the original stochastic accumulator model. Much as evidence accumulation models in perceptual decision making have been adapted to accommodate different task demands, we asked whether the same underlying accumulation process could support both unconstrained action generation and precisely timed self-initiated behaviour through context-dependent modulation of its parameters.

Our simulations are agnostic about how urgency and precision are acquired during learning across trials, and characterise only the within-trial accumulation process to account for varying urgency constraints (target time) and motor plan precision. Within this scope, our modelling shows that a baseline shift in the accumulator can capture urgency-related modulations as reflected in contralateral motor beta power, and a dynamic, target time-locked input modulation can in turn recapitulate the precision-related steepening of the RP. However, an exhaustive model comparison is beyond the scope of this paper, and future work is needed to thoroughly compare this extended version of the model with other plausible alternatives. It is possible that simultaneous adjustments to input strength and distance-to-bound coexist, and that these may be deployed in a task-dependent manner.

Our study has some limitations. First, participants learned target times very quickly, with participants’ performance plateauing around the fourth trial across conditions. This means that only about three trials per block were fully exploratory, and identifying changes in neural signatures over the course of a single block was, therefore, not feasible due to low trial count. Nevertheless, our probabilistic feedback regime ensured considerable trial-by-trial variability in waiting times in both reward window conditions, allowing us to use all trials to assess how motor plan precision influenced motor preparation markers. Second, our study had a limited number of repetitions per condition (20 trials for each target time × reward window condition). However, our RP analyses make use of single trial data, partially mitigating this limitation. In the case of beta power, the analysis tested for a linear scaling of beta power across target time conditions, therefore, imposing strict conditions for significance that render spurious results unlikely, and making use of all available data for the analysis. Future work will aim to replicate the current study and overcome some of its current limitations. A further limitation of our modelling is that the within-trial accumulator is given the learned target waiting time as an exogenous parameter; we do not model the upstream mechanism by which the brain estimates elapsed time and triggers the input modulation at the appropriate moment. A range of candidate timing mechanisms have been proposed (Paton & Buonomano, 2018), and integrating an explicit timing mechanism with the within-trial accumulator is a natural direction for future work. Our study leaves open questions for future research. In this study, we have chosen to refer to the frontocentral negativity preceding self-paced actions as the RP based on the research tradition we follow (Libet et al., 1983). However, a closely related signal warrants consideration. The contingent negative variation (CNV) is a frontocentral negativity with a similar topography and dynamics to the RP and is typically studied in tasks where participants are cued to wait for the appearance of a stimulus (Praamstra et al., 2006). Both the RP and the CNV have been linked to temporal anticipation processes (Alexander et al., 2016; van Rijn et al., 2011), but the extent to which these two signals may play different functional roles and be more or less directly linked with motor initiation remains unclear (see Alexander et al., 2016; Schurger et al., 2021 for discussion). Future work is needed to address these questions in further detail. Finally, we acknowledge that some readers may take issue with the fact that we refer to the frontocentral negativity preceding actions in our task as a Readiness Potential. That is because the foundational RP studies defined the signal not only on the basis of its topography, but also as a function of the actions it precedes, which in those tasks were spontaneous, irregularly timed, and not preplanned actions (Kornhuber & Deecke, 1965; Libet et al., 1983). Since in our task actions were not capricious but rather planned in order to reproduce a specific timing, one might argue that the premotor signals in this study are not an RP proper, but rather an RP-like signal. We have chosen to stick to the conventional label for the sake of simplicity, and because we believe the similarities with conventional tasks (absence of imperative external go cues, internal timing, and self-paced nature of actions) are sufficient to warrant a direct comparison. However, we leave the choice of words to the readers’ discretion.

Overall, our study provides new insight into the neural mechanisms that allow people to precisely control their action timing. As people learn when to act, the initial state of an action preparation process, as indexed by motor beta power, varies systematically with the required waiting time. We replicated the common finding that beta power drops to a common, minimal level at action initiation (Pfurtscheller & Lopes da Silva, 1999). However, the *initial* level of beta power depended strongly on the timing requirements in each block of our task. Earlier and later actions are accompanied by lower and higher initial levels of beta power, respectively, consistent with a strategic adjustment of the starting state of motor preparation. Next, our results on the readiness potential are consistent with a process whereby precise motor planning is accompanied not only by the baseline activity modulations associated with beta power, but also by strong, precisely timed input to a noisy accumulator. This input is distinct from the ongoing stochastic noise assumed in classical accumulator models (Schurger et al., 2012). Whereas noise provides weak, untimed fluctuations that must eventually accumulate to threshold—barring high leakage—the driving input we infer provides a temporally precise signal that modulates the build-up rate of the noisy accumulator when the correct time to act has been learned.

In sum, our results and simulations suggest a plausible neurally informed computational account of the mechanisms potentially underpinning precise action initiation. Our results reconcile stochastic accumulation as a generative model for the RP with a neural architecture that affords flexible, voluntary control of action timing.

## Supplementary Material

Supplementary Material

## Data Availability

All data and analysis code associated with this study are openly available on the Open Science Framework at https://osf.io/26fm4/.
