# Supplementary material for: Distinct motor preparatory signals correlate with coarse vs fine control of self-paced action timing
Source: Imaging Neurosci (Camb). 2026 Nov 4;4:IMAG.a.1434. doi: 10.1162/IMAG.a.1434 (PMC13736801; doi:10.1162/IMAG.a.1434)
Supplement: Supplementary file 1 [file IMAG.a.1434_supp.pdf]

Distinct motor preparatory signals correlate with coarse *vs* fine control of self-paced action timing

Silvia Seghezzi[1,2]*, Elisabeth Parés-Pujolràs*[1,3] and Patrick Haggard[1].

[1] Institute of Cognitive Neuroscience, University College London, London WC1N 3AZ, UK
[2] Birkbeck University of London, London WC1E 7HX
[3] University College Dublin, Belfield, Dublin 4, Ireland

* Silvia Seghezzi and Elisabeth Parés-Pujolràs equally contributed to this work.

Correspondence:
Patrick Haggard, Institute of Cognitive Neuroscience, London, WC1N 3AZ, UK
Email: p.haggard@ucl.ac.uk

Supplementary Materials

Supplementary note 1

|δWT|

The mixed-effects model analysis on |δWT| revealed a significant effect of reward window on |δWT| ($X^2(1)$= 169.06, p<0.0001), indicating greater |δWT| values for the narrow reward window condition. We found a significant effect of Trial number on |δWT| ($X^2(1)$= 510.56, p<0.0001), indicating greater |δWT| values for smaller trial numbers. The effect of Target time was also significant ($X^2(4)$= 80.07, p<0.001), greater |δWT| values for longer Target times.

The two-way interaction between Reward window and Trial number was significant ($X^2(1)$= 13.56, p=0.0002), revealing a steeper slope of the effect of trial number on |δWT| in the narrow reward window condition compared to the wide reward window condition. See Figure **2A**.

The two-way interaction between Target time and Trial number was significant ($X^2(4)$= 22.06, p=0.0002). All slopes of the effect of Trial number on |δWT| were statistically significant when tested independently for each target time (2250: $X^2(1)$= 205.5, p<0.0001, 3500 $X^2(1)$= 100.22, p<0.0001, 4750: $X^2(1)$= 50.27, p<0.0001, 6000 $X^2(1)$= 85.08, p<0.0001, 7250 $X^2(1)$= 87.64, p<0.0001). Post-hoc comparisons revealed a significant difference between the slope of the effect of Trial Number on |δWT| at 2250 and 4750 Target time (t(4299) = -4.66, p < .0001). All other comparisons were not significant after correction for Tukey method (p>0.07). See Figure **2B**.

The two-way interaction between Reward window and Target time was not significant ($X^2(4)$= 1.57, p=0.82). The three-way interaction between Reward window, Target time and Trial number was also not significant ($X^2(4)$= 7.54, p=0.11).

δWT

The mixed-effects model analysis on δWT revealed a significant main effect of Reward window on signed δWT ($X^2(1)$ = 20.19, p < 0.0001), indicating greater δWT values for the narrow reward window condition. The main effect of Signed Previous Feedback was highly significant ($X^2(2)$ = 3510.33, p < 0.0001), confirming that participants adjusted their response times systematically based on the type of feedback received: too early, perfect, or too late. In contrast, Target Time alone did not significantly affect signed δWT ($X^2(4)$ = 0.46, p = 0.977).

The interaction between Reward window and Signed Previous Feedback was significant ($X^2(2)$ = 12.36, p = 0.0021), indicating that participants adjust their behaviour in a manner consistent with the feedback provided. Specifically, when the feedback indicated "too early," participants extended their response time (positive δWT), while "too late" feedback led to shorter response times (negative δWT), however this was marked by a more linear trend in the narrow reward window condition. See Figure **2C**.

The interaction between Target Time and Signed Previous Feedback was also significant ($X^2(8)$ = 81.99, p < 0.0001).

Finally, the three-way interaction among Reward window , Target Time, and Signed Previous Feedback reached significance ($X^2(8)$ = 18.41, p = 0.0183), indicating that the combined effects of reward window and target time modulated how participants adjusted their response times based on the type of feedback they received. See Figure **2D**.

|Timing error|

The model exploration revealed a significant effect of reward window on |Timing error| ($X^2(1)$= 80.10, p<0.0001), indicating greater |Timing error| values for the narrow reward window condition. We found a significant effect of Trial number on |Timing error| ($X^2(1)$= 1179.55, p<0.0001), indicating greater |Timing error| values for smaller trial numbers. The effect of Target time was also significant ($X^2(4)$= 1120.52, p<0.0001), indicating greater |Timing error| values for longer Target times.

The two-way interaction between Reward window and Target time was significant ($X^2(4)$= 77.37, p<0.0001), revealing a significantly greater |Timing error| values for the wide than narrow reward window condition at 4750 target time (z = 2.57, p=0.01), 6000 target time (z = 6.02, p<0.0001) and 7250 target time (z = 10.68, p<0.0001).

The two-way interaction between Reward window and Trial number was significant ($X^2(1)$= 9.59, p=0.002), indicating a steeper slope of the effect of Trial number on |Timing error| for the wide reward window condition than narrow reward window condition.

The two-way interaction between Target time and Trial number was significant ($X^2(4)$= 157.05, p<0.001).

The three-way interaction between Reward window , Target time and Trial number was also significant ($X^2(4)$= 16.56, p=.002). Post-hoc tests revealed a significant steeper slope of the effect of Trial Number on |Timing error| in the wide than narrow reward window condition only at 7250 Target time (t(4855) = 4.78, p < .0001) after correction for multiple comparisons (Tukey method). The slope of Trial Number on |Timing error| was statistically significant in all models (smaller p value<0.0001) when tested separately for Reward window and Target time. See Figure **2E**.

Waiting times

The model exploration revealed a significant effect of reward window on Waiting times ($X^2(1)$= 115.39, p<0.0001), indicating greater Waiting times values for the narrow reward window condition (M=4582.10, SD=186.40) than wide reward window condition (M=4273.46, SD=271.00). We found a significant effect of Trial number on Waiting times ($X^2(1)$= 235.79, p<0.0001), indicating greater Waiting times values for greater trial numbers. The effect of Target time was also significant ($X^2(4)$= 8388.70, p<0.0001), indicating greater Waiting times for longer Target times.

The two-way interaction between Reward window and Target time was significant ($X^2(4)$= 97.59, p<0.0001), revealing significantly greater Waiting times for the narrow than wide reward window condition at 4750 target time (t(4855)=-5.12, p<0.0001), 6000 target time (t(4855)=-7.84, p<0.0001) and 7250 target time (t(4855)=-11.11, p<0.0001).

The two-way interaction between Target time and Trial number was significant ($X^2(4)$= 782.12, p<0.001).

The two-way interaction between Reward window and Trial number was not significant ($X^2(1)$= 1.10, p=0.30).

The three-way interaction between Reward window , Target time and Trial number was significant ($X^2(4)$= 21.15, p=0.0003). Post-hoc tests revealed a significant steeper slope of the effect of Trial Number on Waiting times in the wide than narrow reward window condition only at 7250 Target time (t(4855) = 4.78, p < .0001) after correction for multiple comparisons (Tukey method). The slope of Trial Number on Waiting times was statistically significant in all models (smaller p value<0.0001) when tested separately for Reward window and Target time. See Figure **2F**.

Feedback

The model exploration revealed a significant main effect of Reward window ($X^2(1) = 351.77$, $p < 0.0001$), indicating that the probability of a positive feedback response was higher in the wide compared to the narrow reward window conditions. Similarly, Target time had a significant effect ($X^2(4) = 45.21$, $p < 0.0001$), demonstrating that feedback rates varied across the different target times. In addition, Trial number was a strong predictor of feedback ($X^2(1) = 269.55$, $p < 0.0001$), suggesting that the likelihood of receiving positive feedback systematically increased over the course of the experiment.

The interaction between Reward window and Target time was also significant ($X^2(4) = 17.77$, $p = 0.001$), revealing that the effect of Target time on feedback was modulated by reward window condition. Post hoc test revealed that the probability of a positive feedback response was higher in the wide compared to the narrow reward window condition in every Target time (all p values < 0.0001). See Figure **2G**.

The interaction between Target time and Trial number was significant ($X^2(4) = 23.47$, $p = 0.0001$). Post hoc analysis revealed a statistically significant difference between 4750 Target time and 7250 ($p=0.0001$). All the slopes were significant when tested individually (all p values < 0.0001).

However, the interaction between Reward window and Trial number did not reach significance ($X^2(1) = 0.37$, $p = 0.54$), nor did the three-way interaction among Reward window , Target time, and Trial number ($X^2(4) = 5.32$, $p = 0.26$).

Supplementary note 2

Readiness Potential (RP)

A linear mixed-effects model predicting RP slope from |δWT|, reward window, and Waiting time was conducted. There were no significant main effects for |δWT| ($\chi^2(1) = 2.15$, $p = 0.14$), reward window ($\chi^2(1) = 0.44$, $p = 0.51$), or Waiting time ($\chi^2(1) = 1.70$, $p = 0.19$). However, the interaction between |δWT| and reward window was significant ($\chi^2(1) = 4.31$, $p = 0.038$).

To further explore the significant interaction between |δWT| and reward window, separate slope analyses were conducted for each reward window condition. For the wide reward window condition, the effect of |δWT| was not significant ($\chi^2(1) = 0.15$, $p = 0.70$). In contrast, for the narrow reward window condition, |δWT| significantly predicted RP slope ($\chi^2(1) = 7.11$, $p = 0.008$), suggesting that increases in |δWT| are associated with changes in RP slope specifically within the narrow reward window condition. This pattern suggests that the temporal resolution of the reward window modulates the sensitivity of RP slope to variations in |δWT|.Neither the interaction between |δWT| and Waiting time ($\chi^2(1) = 0.12$, $p = 0.73$) nor the interaction between reward window and Waiting time ($\chi^2(1) = 0.25$, $p = 0.61$) reached significance, and the three-way interaction among |δWT|, reward window, and Waiting time was also not significant ($\chi^2(1) = 1.93$, $p = 0.17$).

Trial-by-trial dynamics of the |δWT| → RP slope relationship within each reward window condition

The |δWT| × Reward window interaction reported in the main text shows that the relationship between trial-by-trial waiting time updates and RP slope differs between reward window

conditions. A natural follow-up question is whether this relationship is stable across trials within a block, or whether it varies with the phase of learning. In particular, one might expect the |δWT| → RP slope relationship to be present early in wide-window blocks, where feedback may still be informative about motor-plan precision before participants identify a roughly-rewarded waiting time, and to fade later in the block, when small updates within a forgiving reward window no longer reflect precise fine-tuning of the motor plan. This account predicts a |δWT| × trial number (Ntrial) interaction within the wide reward window condition.

We tested this directly by refitting the RP slope model separately for each reward window condition, with |δWT|, Ntrial, and their interaction as fixed effects, and a random intercept for participant.

In the narrow reward window condition, the main effect of |δWT| was significant ($\chi^2(1) = 7.15$, $p = 0.007$), but the |δWT| × Ntrial interaction was not ($\chi^2(1) = 0.06$, $p = 0.81$). The main effect of Ntrial was also not significant ($\chi^2(1) = 0.25$, $p = 0.62$). The |δWT| → RP slope relationship in the narrow condition is therefore stable across the block: smaller |δWT| values are associated with steeper RP slope from early in the block onward.

In the wide reward window condition, neither the main effect of |δWT| ($\chi^2(1) = 0.62$, $p = 0.43$), the main effect of Ntrial ($\chi^2(1) = 2.19$, $p = 0.14$), nor the |δWT| × Ntrial interaction ($\chi^2(1) = 1.49$, $p = 0.22$) was significant. The pattern predicted by the "informative early phase" account — an early |δWT| → RP slope effect that fades with trials — is therefore not detectable in our data.

A feature of the task limit the strength of this null result: behavioural performance stabilises rapidly in both reward window conditions, with |δWT| reaching plateau around the 4th trial of a block (see Fig. 2A,B). The within-block range over which a feedback-informativeness interaction could be expressed is therefore at most ~3 trials per participant per block, restricting statistical power for detecting such an interaction.

We therefore conclude that, within the resolution our task affords, the |δWT| → RP slope relationship is reliably present in the narrow reward window condition throughout the block, and is not reliably present in the wide reward window condition at any phase of the block. The |δWT| × Reward window interaction reported in the main text is best characterised as a difference in the overall presence of the |δWT| → RP slope relationship, rather than as a difference confined to a particular phase of the learning trajectory.

Supplementary Note 3:

Robustness of the |δWT| × Reward window interaction on RP slope

To verify that the central RP slope finding does not depend on the choice of statistical framework, we re-analysed the data using two alternative approaches.

Bayesian mixed-effects analysis. We re-fitted the Eq. 5 model within a Bayesian framework using the brms package (Bürkner, 2017). Continuous predictors and the dependent variable were standardised before fitting; weakly informative default priors were used. The model was run with four chains of 4000 iterations each (1000 warmup, 3000 sampling), yielding 12000 post-warmup posterior draws. The model converged cleanly (all R-hat = 1.00, all bulk effective sample sizes > 4800). The |δWT| × Reward window interaction had a posterior mean of 0.06 (standardised units; 95% credible interval [-0.00, 0.12]), with a posterior probability of 97.0% that the interaction is positive. This Bayesian result is consistent with the frequentist mixed-effects analysis ($\chi^2(1) = 4.31$, $p = 0.038$) reported in the main text.

We additionally attempted to fit a maximal random effects structure (Barr et al., 2013) including correlated random slopes for |δWT|, reward window, and their interaction within participants, using both brms and blme (Chung et al., 2013). The brms maximal model failed to converge (R-hat values exceeding 4 across most parameters; bulk effective sample sizes of 4–11; over 7000 divergent transitions out of 12000 samples). We then attempted to fit the same maximal structure in blme, which allows weakly informative priors on the random-effects covariance to aid estimation. The maximal blme model and three progressively simpler step-down structures (uncorrelated random slopes; random slope for |δWT| only) all failed to converge with degenerate Hessian warnings. Together, these results indicate that the maximal structure is not estimable in our dataset, providing direct empirical support for the Matuschek et al. (2017) argument that parsimonious random effects structures are statistically preferable when maximal structures are not estimable.

Subject-level analysis. To verify that the central RP slope finding does not depend on the choice of mixed-effects modelling, we additionally re-ran the analysis using classical subject-wise statistics. For each participant, we fitted a linear regression of single-trial RP slope on |δWT| separately for each reward window condition, and tested the resulting per-subject coefficients against zero using a one-sample t-test.

In the narrow reward window condition, the per-subject |δWT|–RP slope effect was positive on average (mean coefficient = 3.51×10^{-6}), but the test against zero did not reach conventional significance ($t(23) = 1.61$, $p = 0.12$; 95% CI = [-9.90×10^{-7}, 8.01×10^{-6}]; Shapiro-Wilk $W = 0.96$, $p = 0.38$). In the wide reward window condition, the effect was essentially centred on zero (mean coefficient = -1.17×10^{-6}; $t(23) = -0.34$, $p = 0.73$; 95% CI = [-8.18×10^{-6}, 5.84×10^{-6}]; Shapiro-Wilk $W = 0.96$, $p = 0.35$).

The qualitative direction of the effect is therefore preserved across analytic approaches: a positive |δWT|–RP slope relationship is observed in the narrow but not the wide reward window condition. The per-subject narrow-condition effect is at the borderline of conventional significance, whereas the mixed-effects framework, which uses the data more efficiently through partial pooling (Matuschek et al., 2017), estimates the |δWT| × Reward window interaction at $\chi^2(1) = 4.31$, $p = 0.038$ in the frequentist analysis and with 97.0% posterior probability of being positive in the Bayesian analysis.

Summary. Two analyses that use the hierarchical structure of the data (frequentist lmer and Bayesian brms) detect the |δWT| × Reward window interaction at conventional significance thresholds, while the per-subject analysis is at the borderline of detection. This is the expected pattern when a hierarchical model uses information more efficiently than per-subject aggregation in datasets of this size, through partial pooling of information across participants (Matuschek et al., 2017). The convergence of two independent statistical frameworks (frequentist and Bayesian) on the same conclusion provides robust support for the central RP finding.

Supplementary Figures

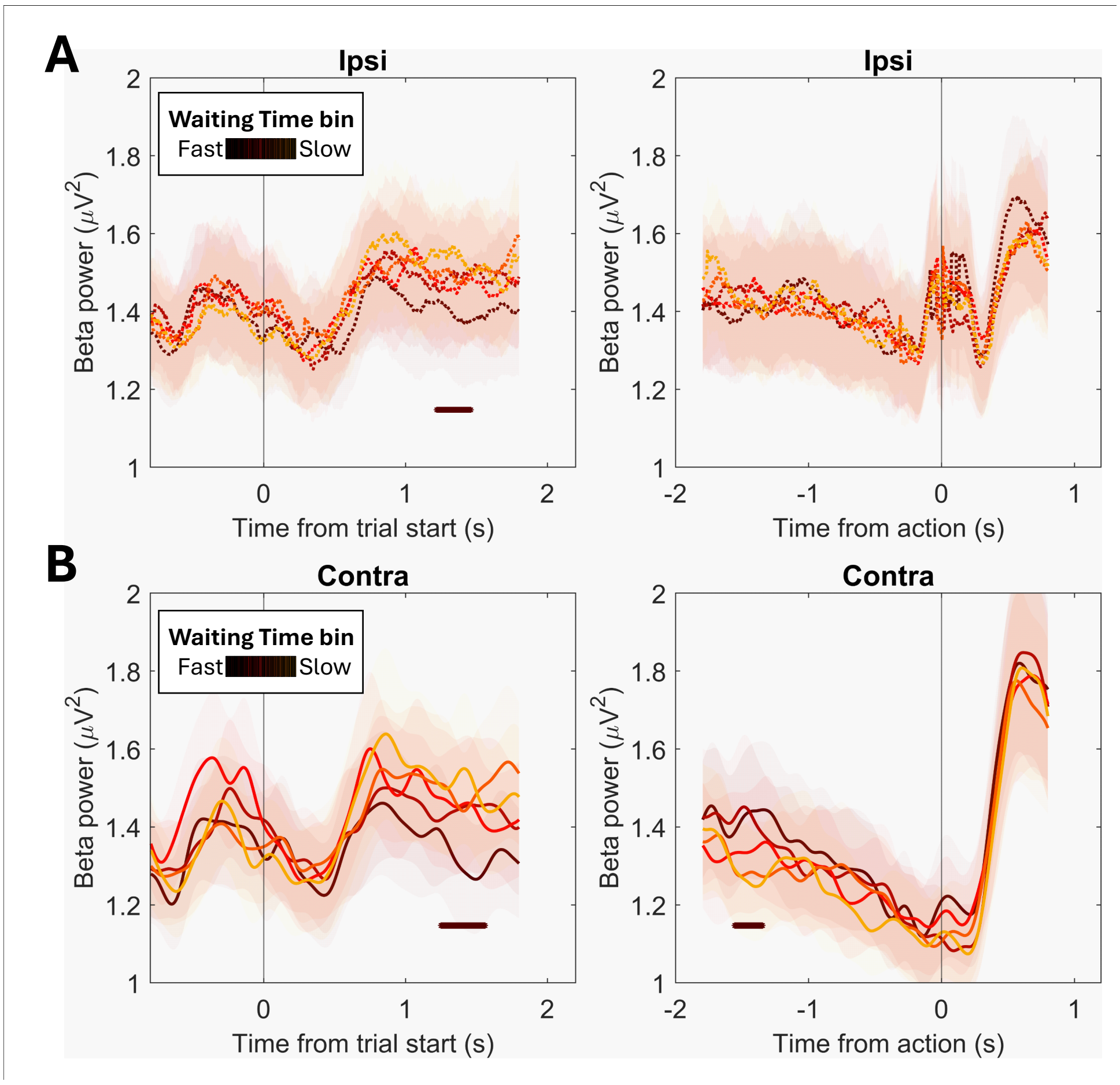

Figure S1. Urgency effects are present in bilateral motor signals. Beta power in Ipsi- [C2, C4] (**A**) and Contra- [C1, C3] (**B**) lateral hemispheres with respect to moving side, sorted by target time and aligned to trial onset (*left*) or action time (*right*). Asterisks represent significant clusters identified following the analysis in Eq. 6.

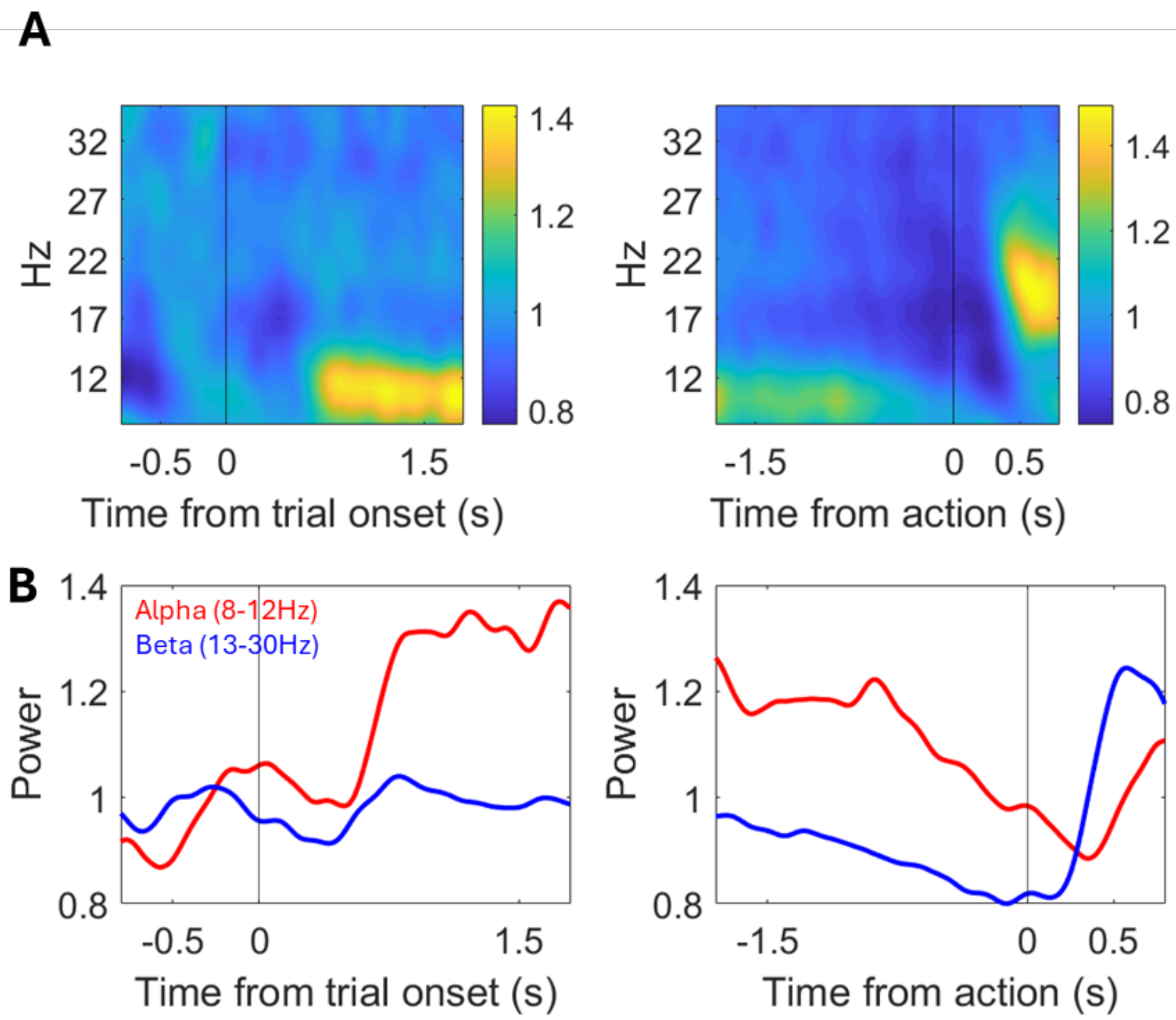

Figure S2. A. Time-frequency spectrograms (8 to 35Hz) aligned to trial onset (*left*) and action (right) on the average of movement-contralateral motor electrodes [C1 & C3]. For visualisation purposes, data were normalised frequency-wise (1Hz resolution) using the 100ms pre-stimulus period. **B.** Time-courses for the alpha (8-12Hz) and beta (13-30Hz) frequency bands, aligned to trial onset (left) and to response (right). Note that the power values in the beta traces in these figures differ slightly from the ones in the manuscript because of the normalisation procedure applied here, which is absent in the manuscript. Alpha band power showed a similar pattern of gradual power decrease from trial start, with slight differences in timing and a stronger initial increase in power at trial onset compared to the beta frequency band. The response aligned traces show the classic event-related desynchronization followed by the post-movement beta rebound.

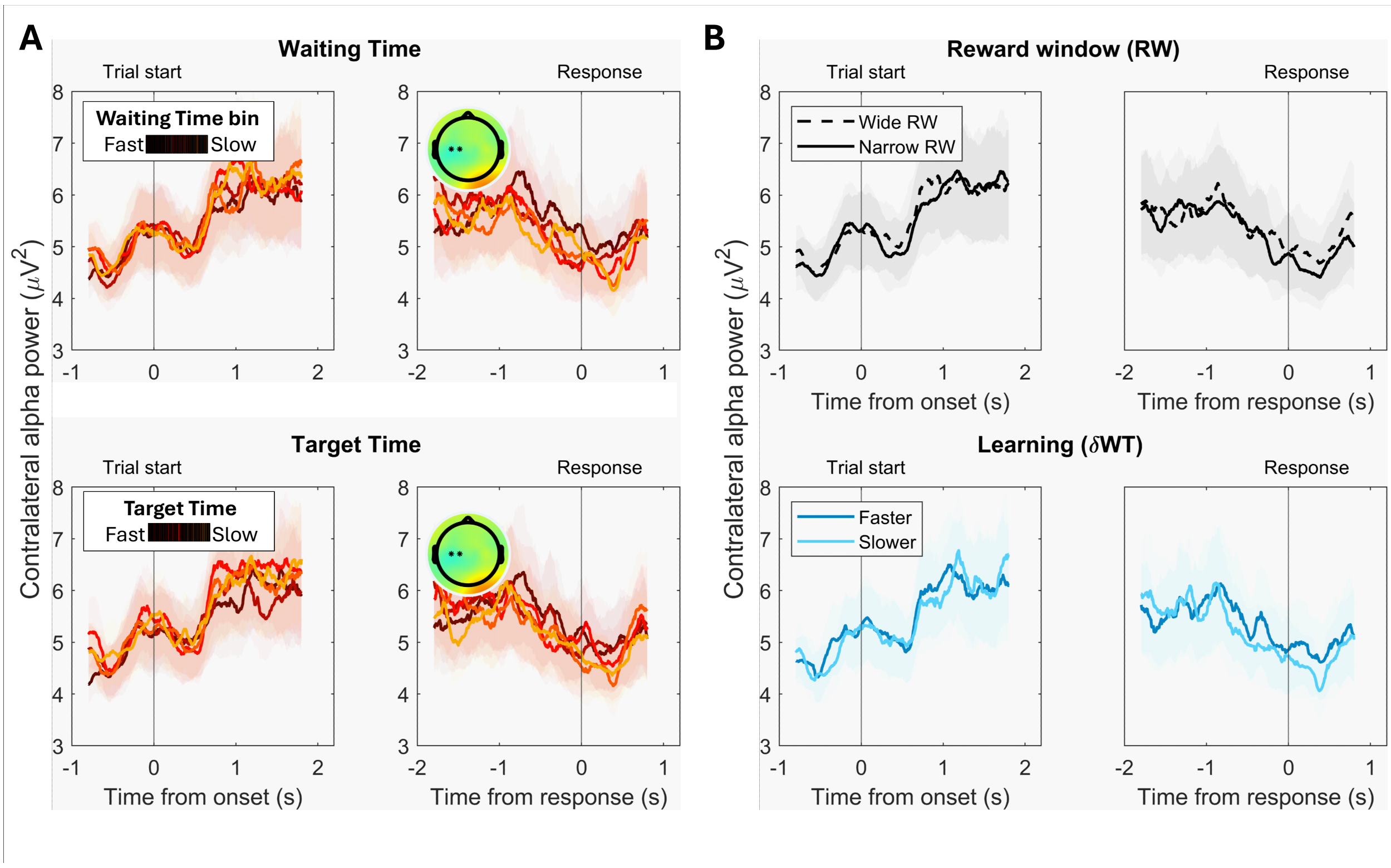

Figure S3. Reproduces the analyses in Fig. 4, but for contralateral alpha band power (8-12Hz). **A.** Contralateral alpha band power, sorted by waiting (*top*) or target time (*bottom*)and aligned to trial onset (*left*) or response (*right*). Analysis from Eq. 6 found no significant clusters indicating a linear relationship between either waiting or target time and alpha power around trial onset. This stands in contrast to results in the beta band **B.** No significant effects of reward window condition (*top*) or learning (*bottom*) were observed, as was the case for the beta band analyses in Fig. 4B.

References

Barr, D. J., Levy, R., Scheepers, C., & Tily, H. J. (2013). Random effects structure for confirmatory hypothesis testing: Keep it maximal. *J Mem Lang*, *68*(3). https://doi.org/10.1016/j.jml.2012.11.001

Bürkner, P.-C. (2017). brms: An R package for Bayesian multilevel models using Stan. *Journal of statistical software*, *80*, 1-28.

Chung, Y., Rabe-Hesketh, S., Dorie, V., Gelman, A., & Liu, J. (2013). A nondegenerate penalized likelihood estimator for variance parameters in multilevel models. *Psychometrika*, *78*(4), 685-709.

Matuschek, H., Kliegl, R., Vasishth, S., Baayen, H., & Bates, D. (2017). Balancing Type I error and power in linear mixed models. *Journal of memory and language*, *94*, 305-315.
